# Intracellular fraction of zona pellucida protein 3 is required for the oocyte-to-embryo transition in mice

**DOI:** 10.1093/molehr/gaad038

**Published:** 2023-10-31

**Authors:** Steffen Israel, Julia Seyfarth, Thomas Nolte, Hannes C A Drexler, Georg Fuellen, Michele Boiani

**Affiliations:** Max Planck Institute for Molecular Biomedicine, Department of Cell & Tissue Dynamics, Muenster, Germany; Max Planck Institute for Molecular Biomedicine, Department of Cell & Tissue Dynamics, Muenster, Germany; Max Planck Institute for Molecular Biomedicine, Department of Cell & Tissue Dynamics, Muenster, Germany; Max Planck Institute for Molecular Biomedicine, Department of Cell & Tissue Dynamics, Muenster, Germany; Rostock University Medical Center, Institute for Biostatistics and Informatics in Medicine and Aging Research (IBIMA), Rostock, Germany; Max Planck Institute for Molecular Biomedicine, Department of Cell & Tissue Dynamics, Muenster, Germany

**Keywords:** animal model, embryo development, oocyte, *Trim-away*, zona pellucida

## Abstract

In oocyte biology, the zona pellucida has long been known to operate three extracellular functions downstream of the secretory pathway, namely, encasing the oocytes in ovarian follicles, mediating sperm–oocyte interaction, and preventing premature embryo contact with oviductal epithelium. The present study uncovers a fourth function that is fundamentally distinct from the other three, being critical for embryonic cell survival in mice. Intriguingly, the three proteins of the mouse zona pellucida (ZP1, ZP2, ZP3) were found abundantly present also inside the embryo 4 days after fertilization, as shown by mass spectrometry, immunoblotting, and immunofluorescence. Contrary to current understanding of the roles of ZP proteins, ZP3 was associated more with the cytoskeleton than with secretory vesicles in the subcortical region of metaphase II oocytes and zygotes, and was excluded from regions of cell–cell contact in cleavage-stage embryos. *Trim-away*-mediated knockdown of ZP3 in fertilized oocytes hampered the first zygotic cleavage, while ZP3 overexpression supported blastocyst formation. Transcriptome analysis of ZP3-knockdown embryos pointed at defects of cytoplasmic translation in the context of embryonic genome activation. This conclusion was supported by reduced protein synthesis in the ZP3-knockdown and by the lack of cleavage arrest when *Trim-away* was postponed from the one-cell to the late two-cell stage. These data place constraints on the notion that zona proteins only operate in the extracellular space, revealing also a role during the oocyte-to-embryo transition. Ultimately, these data recruit ZP3 into the family of maternal factors that contribute to developmental competence of mouse oocytes.

## Introduction

Two of the preconditions for correct embryonic development are that oocytes are protected from physical injury and become fertilized by a single spermatozoon. In mammals, these preconditions are secured by the zona pellucida, an extracellular glycoprotein coat encoded by either three or four single-copy genes in placental mammals (e.g. ZP1–3 in mice, ZP2–4 in pigs and cows, ZP1–4 in humans (Conner *et al.*, 2005) with variations in nomenclature) and by additional genes in marsupials (Moros-Nicolas *et al.*, 2021). We are going to use the term ‘zona pellucida’ (‘zona’ for short) to indicate the cytological structure (the extracellular coat), and the acronym ZP to indicate the specific molecular players (genes, transcripts, or proteins). During mouse oogenesis, the transcription of ZP genes reaches maximum in midsized oocytes (50–60 μm in diameter), before declining to 5% of peak values in ovulated oocytes (Roller *et al.*, 1989; Epifano *et al.*, 1995). ZP genes are not transcribed after fertilization. The immediate translation products are pro-peptides, which are glycosylated and include a signal peptide for routing to the secretory pathway of the oocyte (Roller *et al.*, 1989; Epifano *et al.*, 1995). Along the secretory pathway ZP proteins colocalize in the endoplasmic reticulum and Golgi complex (Hoodbhoy *et al.*, 2006), where they undergo further post-translational modifications. Owing to these modifications, the molecular weights of each of the three ZP proteins vary, for example: ZP3 weighs 44 kDa as a naked polypeptide chain and up to 83 kDa as a glycosylated chain (79 kDa according to Boja *et al.* (2003); 80 kDa according to Salzmann *et al.* (1983); 81 kDa according to Shi *et al.* (2004); 83 kDa according to Bleil and Wassarman (1980) and Shimizu *et al.* (1983)). Once the ZP proteins have been secreted and assembled together, they build the zona that surrounds the embryos until the blastocyst stage, aided by the long half-life of its proteins (>100 h; Shimizu *et al.*, 1983).

**Figure 1.**
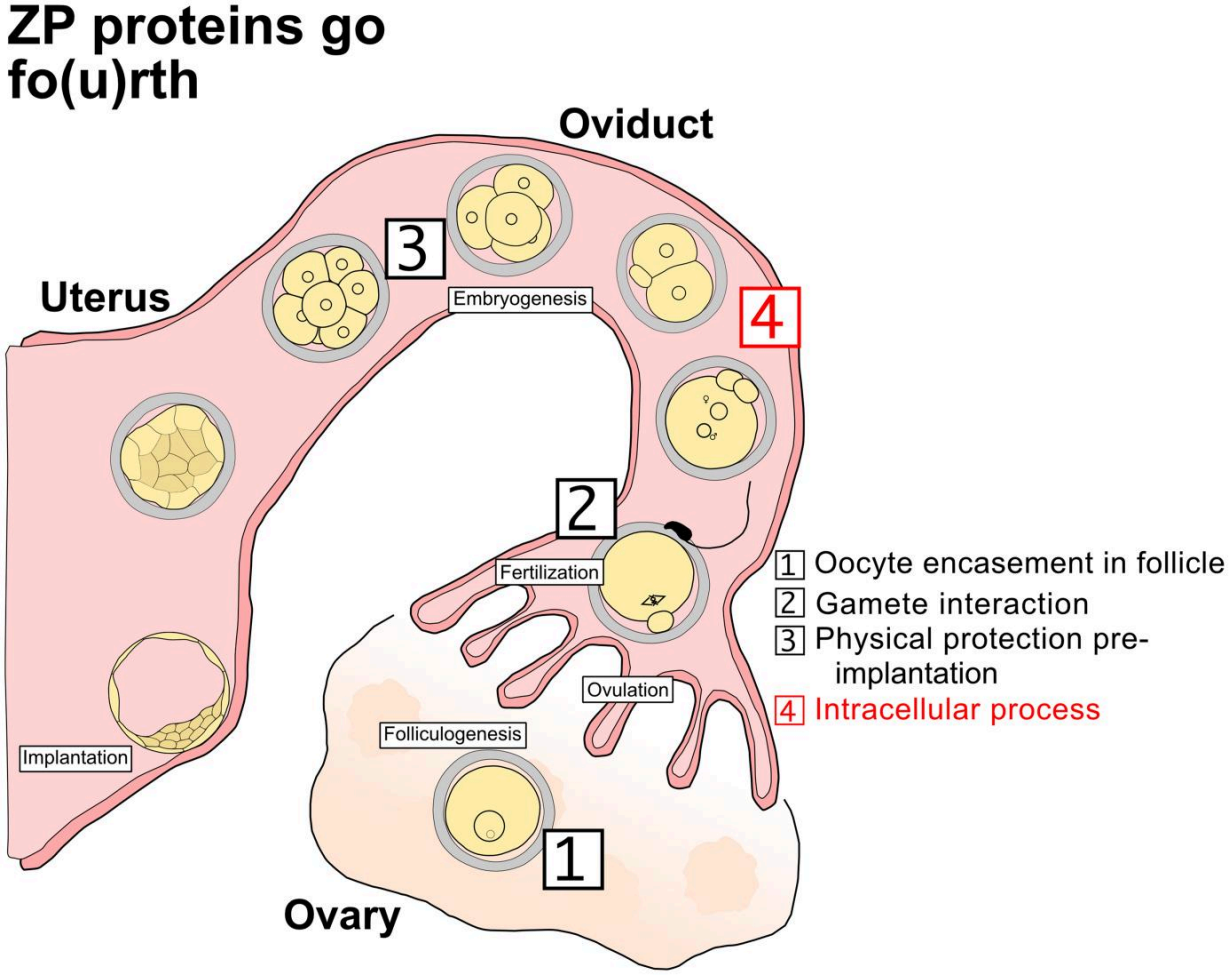
**Schematic summary of the main findings of this study of intracellular zona pellucida protein 3 and its newly discovered function in early mouse embryos.** ZP, zona pellucida.

The current model of the zona builds on the results of ZP mutagenesis studies conducted mostly in mice (Liu *et al.*, 1996; Rankin *et al.*, 1996, 1999, 2001) but also in rabbits and rats (Lamas-Toranzo *et al.*, 2019; Zeng *et al.*, 2021). Based on the mutant phenotypes of ZP2−/− and ZP3−/− oocytes, the current model predicates that the zona operates its functions exclusively in the extracellular space. In chronological order, these functions are, as pictured in Fig. 1: to encase the oocyte in the follicle while permitting exchange with the surrounding environment (e.g. follicular cells) via transzonal projections and microvilli; to mediate species-specific monospermic fertilization; and to hold the blastomeres together before compaction while protecting them from premature physical contact with the oviductal epithelium (reviewed in Wassarman and Litscher (2022)). As a particular case of the encasement, the ZP proteins may also operate as scaffolds through which transzonal projections and possibly microvilli transduce mechanochemical forces that organize and stabilize the oocyte cytoskeleton in its regions, such as the subcortical region (Ouni *et al.*, 2020; Fiorentino *et al.*, 2023). In turn, ZP mutations are held responsible for empty follicle syndrome and polyspermic oocyte fertilization (Fei and Zhou, 2022; Sun *et al.*, 2023).

A limitation of the above model is that the direct consequences of the fertilization defects, for example polyploidy, overshadowed other possible effects of ZP mutations on embryonic processes. Since the ZP2−/− and ZP3−/− oocytes lacked the zona and thereby an effective block to polyspermy (Rankin *et al.*, 2001), they produced polyploid embryos, which suffer developmental defects regardless of any hypothetical roles played by the ZP proteins after fertilization. To obviate this limitation, investigators fertilized the mutant ZP2−/− and ZP3−/− oocytes with a reduced concentration of wild-type sperm *in vitro* (commensurate with the lack of the zona), and selected the oocytes that had been fertilized monospermically (Rankin *et al.*, 2001). As a result, the zygotes were diploid and presumably contained one ZP2 or ZP3 allele from the father, although the general consensus is that ZP genes are not expressed in embryogenesis. When these zygotes were cultured to blastocyst and transferred to the uterus (Rankin *et al.*, 2001), birth rates were still severely reduced compared to controls whose zonae had been removed manually (Rankin *et al.*, 2001; Fan *et al.*, 2022). The reduced competence of ZP−/− oocytes to form viable blastocysts after monospermic fertilization can be explained in two, non-mutually exclusive ways: perduring of mutant oocytes’ defects in the embryo; additional and hitherto uncharacterized functions of ZP2 or ZP3 in the embryonic cytoplasm.

We reasoned that in order to resolve the issues of perduring oocyte defects versus new functions of the ZP proteins in the embryo, a postfertilization approach would be beneficial. In plain words, it would be necessary to alter the embryo, with respect to ZP, without altering the precursor oocyte. Since the ZP genes are transcriptionally silenced by the end of oogenesis, a suitable molecular approach is one that hinges on proteins, such as the immunological method of TRIM21-mediated proteasomal degradation, briefly known as ‘*Trim-away*’ (Clift *et al.*, 2017). In this study, as we checked how much ZP proteins were present in mouse oocytes, we realized that they were much more abundant than commonly thought, and they also endured throughout preimplantation development. Blastocysts manually freed of the zona still contained almost as much internal ZP proteins as zona-intact blastocysts. We performed *Trim-away* of ZP proteins in zygotes, observing that knockdown of ZP3 and ZP2 prevented the first and the second cleavage, respectively, while knockdown of ZP1 resulted in stunted blastocyst formation. We therefore focused on ZP3 given its earlier and stronger knockdown phenotype compared to that of ZP1 and ZP2. ZP3 protein was concentrated in the subcortical region of metaphase II (MII) oocytes and zygotes but was excluded from regions of cell–cell contact in cleavage-stage embryos, resembling the protein distribution of the subcortical maternal complex (SCMC; Li *et al.*, 2008). Notably, subcortical ZP3 was refractory to extraction with detergent Triton X-100, but underwent centripetal and lateral relocation upon, respectively, oocyte culture in medium containing the microtubule disruptor nocodazole or blastomere culture in medium lacking Ca(2+) ions. This observation prompted us to apply nocodazole and latrunculin B during *Trim-away*, without, however, resulting in enhanced knockdown of ZP3. These effects support a physical connection of ZP3 with the cytoskeleton rather than with secretory membranes. Transcriptomic comparison of ZP3-knockdown zygotes versus normal counterparts revealed defects of cytoplasmic translation in the context of embryonic genome activation (EGA). Collectively, this new information supports that ZP proteins are required for developmental competence not only outside but also inside mature oocytes, where the ZPs clearly do more than interacting with each other to form a secreted coat that serves as a scaffold for, for example, transzonal projections, that mediates gamete interaction, and that holds the blastomeres together. We propose that ZP3 protein also serves an additional function as a novel intracellular player in the oocyte-to-embryo transition (Fig. 1), thereby adding a new recruit to the family of maternal factors that contribute to the developmental competence of oocytes (Innocenti *et al.*, 2022).

## Materials and methods

### Mouse embryo production

All mice used in this study (N ≈ 250 females B6C3F1, N ≈ 50 males CD1 over a period of ≈1 year) were reared in-house at the MPI Münster. They were maintained in individually ventilated type 2 l cages (EHRET GmbH Life Science Solutions, 79111 Freiburg, Germany), with autoclaved Aspen wood as bedding material and a cardboard tube as enrichment, in groups of five females or individually as males. Access to water (acidified to pH 2.5) and food (Teklad 2020SX, ENVIGO RMS GmbH, Düsseldorf, Germany) was *ad libitum*. The animal room was maintained at a controlled temperature of 22°C, a relative humidity of 55%, and a 14/10 h light/dark photoperiod (light on at 6:00 a.m.). The hygiene status was monitored every 3 months, as recommended by Federation of European Laboratory Animal Science Associations (FELASA), and the sentinel mice were found free of pathogens that might have affected the results of this study. Ovulation was induced by i.p. injection of pregnant mare’s serum gonadotrophin (eCG) (Pregmagon, IDT, 06861 Dessau-Roßlau, Germany) and hCG (Ovogest, MSD Tiergesundheit, Germany). Lean B6C3F1 females aged 8–10 weeks and weighing ∼25 g were injected i.p. using a 27G needle, at 5 p.m., with 10 IU eCG and 10 IU hCG 48 h apart. The females were mated to CD1 studs aged 3–12 months to produce zygotes, or left unmated to collect MII oocytes. On the next morning, the females were killed by cervical dislocation. The cumulus–oocyte complexes were recovered, dissociated in hyaluronidase (CAT No. 151271, ICN Biomedicals, USA; 50 IU/ml in HCZB), dissolved in Hepes-buffered Chatot, Ziomek, and Bavister medium (HCZB) with bovine serum albumin (BSA; Probumin, Milllipore, Kankakee, IL, USA) replaced by polyvinylpyrrolidone (PVP, 40 kDa; CAT No. 529504, Calbiochem, EMD Biosciences, La Jolla, CA, USA) 0.1% w/v. The cumulus-free MII oocytes were cultured in 500 µl of α-MEM medium (CAT No. M4526, Sigma-Aldrich Chemie GmbH, Taufkirchen, Germany) supplemented with 0.2% (w/v) BSA, and the cumulus-free zygotes in 500 µl of potassium (K) simplex optimization medium (KSOM) prepared in house as per the original recipe (Summers *et al.*, 2000). KSOM contained free amino acids both essential and non-essential (hence called KSOM(aa)), 0.2% (w/v) BSA and gentamicin (50 IU/ml), and was used in a four-well Nunc plate without oil overlay, at 37°C under 6% CO_2_ in air. To produce parthenogenetic embryos, MII oocytes were activated for 6 h in Ca(2+)-free α-MEM medium containing 10 mM SrCl_2_ and 5 μM Latrunculin B (CAT No. 428020, Merck Millipore, Darmstadt, Germany). Following activation, the pronuclear-stage oocytes were washed in KSOM(aa) in three steps of 10 min each to remove the intracellular Latrunculin B accumulated. Parthenotes were cultured in 4-well plates containing 500 μl KSOM(aa) medium at 37°C (6% CO_2_).

### Stable isotope labeling of embryonic protein synthesis during preimplantation

Fertilized oocytes were retrieved from oviducts after mating, and cultured in KSOM(aa) medium prepared and used as described above (see Mouse embryo production), except that: the albumin was replaced with PVP (0.1% w/v) and the two canonical amino acids L-Arginine (0.3 mM) and L-Lysine (0.2 mM) were replaced with the non-radioactive isotopic forms Arg-10 (^13^C_6_$H_{14}^{15}$N_4_O_2_; CAT No. CNLM-539-H-PK, Cambridge Isotope Laboratories, Tewksbury, MA, USA) and Lys-8 (^13^C_6_$H_{14}^{15}$N_2_O_2_; CAT No. CNLM-291-H-PK, Cambridge Isotope Laboratories). The fertilized oocytes cultured in the presence of Arg-10 and Lys-8 were collected for proteome analysis at the blastocyst stage.

### Transfer of Arg-10 and Lys-8 labeled mouse blastocysts to the uterus

To confirm that the isotopic labeling preserved the developmental potential of the zygotes, groups of eight blastocysts were transferred surgically to one uterine horn of pseudopregnant CD1 recipients that had received the copulation plug from vasectomized CD1 males 2 days prior to the embryo transfer. Prior to surgery, CD1 foster mothers were anesthetized with Ketamine (80 mg/kg body weight)/Xylazin (16 mg/kg)/Tramadol (15 mg/kg) in PBS, delivered i.p. The surgical wounds were sutured with resorbable Marlin violett. Post-surgical pain was alleviated by providing the animals with Tramadol in drinking water (1 mg/ml). Pregnancies were evaluated by C-section just prior to term (embryonic day 18.5).

### Proteome analysis of labeled blastocysts

Following pooling and lysis of the labeled blastocysts in sodium dodecyl sulfate lysis buffer, blastocyst proteins were subjected to rapid acetone precipitation at room temperature in the presence of 20 mM NaCl, according to the method described by Nickerson and Doucette (2020). The air-dried pellet was then further processed using the Preomics iST kit according to the manufacturer’s instructions starting with the resuspension of proteins in 20 µl of the kit’s lysis buffer (Preomics, 82152 Planegg-Martinsried, Germany). The digested and purified sample was subsequently dried in an Eppendorf Concentrator and resuspended in Buffer A (0.1% formic acid) for the liquid chromatography–mass spectrometry (LC-MS/MS) measurement on a Q Exactive HF mass spectrometer online coupled to an EASY nLC 1200 nano-HPLC pump via a Nanospray Flex ion source (Thermo Fisher Scientific, Karlsruhe, Germany). Peptide mixtures were chromatographically separated on a 50-cm long fused silica emitter (CoAnn Technologies, Aarle-Rixtel, The Netherlands) home-packed with 2.6 µm HALO ES-C18 beads (Advanced Material Technology, Wilmington, DE, USA) via a linear gradient from 3% Buffer B (80% acetonitrile, 0.1% formic acid) to 35% B within 220 min, before being ramped to 60% and 98% B within 20 and 3 min, respectively (flow rate 300 nl/min). Data were recorded using a top 17 data-dependent method (scan range 300–1750 m/z; MS1 resolution 60 000; AGC target 3e6; maximum IT 100 ms; MS2 resolution 15 000; AGC target 1e5; maximum IT 50 ms; normalized collision energy = 27 V; dynamic exclusion enabled for 20 s). Raw data were processed for identification and quantification by MaxQuant Software (version 2.0.3.0; Cox and Mann, 2008) with the ‘iBAQ’ option enabled and the ‘requantify’ option disabled. MaxQuant provides intensities for heavy and light labeled peptides and proteins separately and this also pertains to the iBAQ values. The search for identification was performed against the UniProt mouse database (version from April 2019) concatenated with reversed sequence versions of all entries and supplemented with common laboratory contaminants. Parameters defined for the search were trypsin as the digesting enzyme, allowing two missed cleavages, a minimum length of six amino acids, carbamidomethylation at cysteine residues as a fixed modification, oxidation at methionine, and protein N-terminal acetylation as variable modifications. The maximum mass deviation allowed was 20 ppm for the MS and 0.5 Da for the MS/MS scans. Protein groups were regarded as identified with a false discovery rate (FDR) set to 1% for all peptide and protein identifications; at least one unique peptide was required for each protein analyzed further. The molar fractional content of each protein P in a sample (relative iBAQ intensities=riBAQ_P_) was determined for the light and heavy labeled proteoforms independently, according to Shin *et al.* (2013), as follows:


$$\mathrm{riBAQ}=\frac{\mathrm{iBA}Q_{i}}{\sum_{i=1}^{n}\mathrm{iBA}Q_{i}}$$

### Evacuation of zonae for immunoblot analysis

Oocytes were transferred in groups of 10 to a micromanipulation drop on the stage of a Nikon Eclipse TE2000-U inverted microscope fitted with Nomarski optics and Narishige micromanipulator. The micromanipulation medium consisted of HCZB. Each oocyte was held firmly in place with the holding needle by applying negative pressure (suction). An Eppendorf’s TransferTip (ES) needle operated by an Eppendorf’s CellTram Vario (Eppendorf, Hamburg, Germany) was used to pierce through the zona and aspirate the oocyte. The evacuated zonae were concentrated on the bottom of a tube by centrifugation, the supernatant was replaced with RIPA buffer, and the lysate was processed further as described below.

### Loosening of interblastomere contact and isolation of blastomere in two-cell stage embryos

Two-cell embryos were incubated in 0.2 mM D(+) glucose, 0.2 mM pyruvate, 10 mM lactate, 0.5% w/v BSA, in 0.9% w/v sodium chloride, in a suspension petri dish (CAT No. 430588, Corning, NY, USA) on a warm plate set at 37°C. This Ca(2+)-free medium caused the cell–cell contacts to become loose, and the blastomeres to become rounder. When applicable, the sibling blastomeres were extracted from the zona using a TransferTip (ES) needle operated by a CellTram Vario (Eppendorf), as described (Casser *et al.*, 2017). Isolated blastomeres and whole two-cell embryos were processed further as described below.

### Disruption of zygotic cytoskeleton

Zygotes were incubated in nocodazole (5 µg/ml; CAT No. 487928, Calbiochem) and latrunculin B (5 µM), diluted in KSOM(aa) from a 1000× stock solution prepared in dimethylsulfoxide (DMSO). As a control, zygotes were incubated in the solvent of nocodazole and latrunculin B, that is, in DMSO 0.1% (v/v) in KSOM(aa). After 5 h at 37°C under 6% CO_2_ in air, the zygotes were processed further as described in ‘Immunofluorescence analysis of ZP1, ZP2, ZP3, GM130, calreticulin, and tubulin in oocytes and preimplantation embryos’.

### Immunofluorescence analysis of ZP1, ZP2, ZP3, GM130, calreticulin, and tubulin in oocytes and preimplantation embryos

Oocytes or embryos were analyzed by performing an immunostaining followed by confocal microscopy imaging, as per our routine protocol. Briefly, embryos were fixed with 3.7% formaldehyde in 1× PBS for 20 min at room temperature, permeabilized with 0.1% Triton X-100 in 1× PBS for 20 min at room temperature, and then blocked with 2% BSA, 2% glycine, 5% donkey serum, 0.1% Tween 20 in 1× PBS overnight at 4°C. When applicable, the zona was removed by bathing the oocytes or embryos in acidic Tyrode solution prewarmed at 30°C (CAT No. T1788, Sigma-Aldrich Chemie GmbH, Taufkirchen, Germany). The following primary antibodies were applied to the specimens for 2 h at room temperature: anti-ZP1 polyclonal IgG raised in rabbit (CAT No. PA5-101973, Invitrogen, Thermo Fisher Scientific, Karlsruhe, Germany), anti-ZP2 polyclonal IgG raised in rabbit (CAT No. A10126, ABclonal Germany GmbH, Düsseldorf, Germany), anti-ZP3 polyclonal IgG raised in rabbit (CAT No. 21279-1-AP, Proteintech Germany GmbH, Planegg-Martinsried, Germany), anti-ZP3 monoclonal IgG raised in mouse (ATCC IE-10 CRL-2462; 0.5 mg/ml), in dilutions of 1:100, respectively. For immune co-localization analysis after oocyte extraction with Triton X-100, the following primary antibodies were used: mouse monoclonal IgG anti-GM130 (CAT No. 610823, BD Bioscience, Heidelberg, Germany), and rabbit polyclonal IgG anti-Calreticulin (CAT No. PA3-900, Thermo Fisher Scientific, Karlsruhe, Germany). For the association of ZP3 with the cytoskeleton, mouse monoclonal IgG anti α-tubulin (CAT No. T6199, Sigma-Aldrich Chemie GmbH) was used. An appropriate Alexa Fluor-tagged secondary antibody (Invitrogen) was matched to the primary and incubated for 1–2 h at room temperature. DNA counterstaining was performed with YO-PRO-1 or Hoechst 33342 (1 µM). For imaging, embryos were placed in 5 µl drops of PBS on a 50-mm thin-bottom plastic dish (Lumox hydrophilic dish, Greiner Bio-One, Frickenhausen, Germany) and overlaid with mineral oil (CAT No. M8410, Sigma-Aldrich Chemie). Images were captured using a 20× CFI Plan Apochromat VC objective on an inverted motorized Nikon TiE2000 microscope fitted with an Andor Dragonfly 502 spinning disc confocal unit Scanning System. Optical sections per embryo were captured using a high resolution (2048×2048 pixel) sCMOS camera. Maximum projections were analyzed with Fiji (Schindelin *et al.*, 2012). For quantification of ZP3 after cytoskeleton disruption, plot profiles were generated using Fiji by drawing a line along the horizontal diameter of the oocytes, irrespective of the orientation of the animal-vegetal axis. Images were pseudocolored using the ‘Union Jack’ LUT of Fiji.

### Immunoblotting analysis of ZP3 expression in preimplantation mouse embryos

Oocytes or embryos were gently centrifuged in protein-free HCZB medium at 39 *g* for 20 min to form a tiny pellet (Supplementary Fig. S1). The supernatant was carefully aspirated using a mouth-operated micropipette, and replaced by RIPA buffer containing protease inhibitors. The resultant lysates were mixed with 6× Laemmli sample buffer and boiled for 5 min at 99°C. These samples were loaded on a 12% separation gel and blotted onto a PVDF membrane. The membrane was blocked for at least 3 h and incubated (3% nonfat dry milk in 0.1% PBS-Tween 20) with primary antibodies overnight at 4°C. The antibodies against ZP3 (anti-ZP3: CAT No. PA5-89033, Invitrogen; CAT No. 21279-1-AP, Proteintech) were applied at a dilution factor of 1:1000 (ZP3). Signal intensities were standardized on α-tubulin (1:5000, CAT No. T6199, Sigma-Aldrich Chemie GmbH). After 3× washing in 0.1% PBS-Tween 20, the blot was incubated with horse-radish peroxidase (HRP)-coupled secondary antibody at room temperature for 1 h. The membrane was washed and then developed with chemiluminescent HRP substrate solution. The chemiluminescent signal was detected using the AGFA Curix 60.

### Epitope masking and proteasomal degradation of ZP proteins (Trim-away)

In order to mask the protein of interest, zygotes were microinjected with ZP3 antibody (CAT No. 21279-1-AP, Proteintech) together with dextran beads fluorescently labeled with Oregon Green (OGDB; 70 kDa; CAT No. D7173, Thermo Fisher Scientific) at 16 h post-hCG. In order to also degrade the protein, zygotes, early two-cell embryos, and late two-cell embryos were microinjected with a mixture of *mCherry-mTrim21* mRNA, OGDB, and ZP1 (CAT No. PA5-101973, Invitrogen), ZP2 (CAT No. A10126, ABclonal), or ZP3 antibody (CAT No. 21279-1-AP, Proteintech; CAT No. PA5-89033, Invitrogen; ATCC IE-10 CRL-2462) at ∼16-, 40-, and 48-h post-hCG, respectively: concentrations in the mixture were 0.15 mg/ml mRNA, 0.017 mg/ml OGDB, and 1 mg/ml antibody, respectively, in MilliQ water. The mRNA was purified with Quick-RNA MicroPrep (CAT No. R1051, Zymo Research, 79110 Freiburg, Germany) and preserved in MilliQ water at −80°C. The antibody was washed three times and concentrated at 4°C using Amicon Ultra-0.5 100-kDa centrifugal filter devices (CAT No. UFC100, Merck Millipore, Darmstadt, Germany), which remove salts and preservatives (e.g. sodium azide) and stabilizers (e.g. BSA), replacing most of the antibody buffer with water. This buffer was collected as flow-through of the Amicon device and set aside for later use as a control. Microinjection of the mRNA–antibody–OGDB mixture was conducted on the stage of a Nikon TE2000U microscope fitted with a piezo drill (PrimeTech, Ibaraki, Japan), using a blunt-end glass needle (inner diameter 6–7 microns, outer diameter 8–9 microns) filled with 2–3 µl mercury at the tip. Volumes were pressure-injected into the zygote or blastomere using a Gilmont GS-1200 micrometer syringe operated manually. During the microinjection, cells were kept in a 200- to 300-µl drop of HCZB medium on a glass-bottomed (Nomarski optics) dish at a room temperature of 28°C. After microinjection, zygotes and two-cell embryos were allowed to recover in the drop for 5–10 min, before returning them to KSOM(aa) medium. For the *Trim-away* depletion of ZP3 operated in conjunction with nocodazole treatment, the zygotes that recovered from the microinjection were cultured in KSOM(aa) medium containing nocodazole (5 µg/ml) and latrunculin B (5 µM) for 5 h.

### Synthesis of mRNA for microinjection

For *Trim-away*, an *ZP3-eGFP* expression construct built on plasmid mouse CENP-B-EGFP was a gift from Michael Lampson (Addgene Plasmid No. 107264; http://n2t.net/addgene:107264; RRID: Addgene_107264). For ZP3 overexpression, the coding sequence of ZP3 (NM_011776.1) was substituted for that of Cenpb in the plasmid CENP-B-EGFP. The construct is apt to support strong transcription given the presence of Kozak 5′, *Xenopus* 5′ Globus, and *Xenopus* 3′-UTR sequences. For *in vitro* transcription, plasmids were linearized with SwaI (CAT No. FD1244, Thermo Fisher Scientific). Capped mRNA was synthesized with T7 polymerase (Ambion mMessage mMachine T7 kit) according to the manufacturer’s instructions.

### Transcriptome analysis of embryos sampled 24 h after ZP3 knockdown

To assess the consequences of ZP3 inactivation on embryonic gene expression, we generated an early transcriptomic dataset at 10 h (one-cell stage) and a late transcriptomic dataset at 24 h (chronological two-cell stage). In the early dataset, samples comprised 13 embryos in each of two groups: group 1 injected with *mCherry-Trim21* mRNA, OGDB, and buffer of the ZP3 Proteintech antibody (triplicate), and group 2 injected with *mCherry-Trim21* mRNA, OGDB, and anti-ZP3 Proteintech antibody (triplicate). In the late dataset, next to these two groups, one additional sample of embryos without any treatment was also included as an outgroup comparison, and the samples comprised 10 embryos each. Total RNA was extracted and purified using Quick-RNA MicroPrep (Zymo Research). The library preparation of the total RNA was performed with the NEBNext^®^ Single Cell/Low Input RNA Library Prep Kit for Illumina (CAT No. NEB No. E6420S/L, New England Biolabs GmbH, Frankfurt am Main, Germany). Single-read sequencing with a read length of 72 bp was performed on Illumina’s NextSeq^®^ 2000 System using the corresponding NextSeq2000 P3 Reagent Kit. Total RNA integrity and quality of the library were assessed using a TapeStation4200 (Agilent, Santa Clara, CA, USA). On average, the libraries contained 18.4±3.6 million 72-base-single-end reads. Using a molecular barcode, the samples were demultiplexed and converted to fastq data using bcl2fastq v3.8.4 (Illumina) quality controlled (FastQC; Andrews, 2010; 8 January 2019: Version 0.11.9 released). Trimmomatic was used for adapter trimming and read filtering (Bolger *et al.*, 2014). The resulting reads were aligned to the reference genome (*Mus musculus* Ensembl GRCm38) using Hisat2 (Kim *et al.*, 2015). The aligned reads were sorted using samtools (Li, 2011). The sorted and aligned reads were counted into genes using htsec-counts (Anders *et al.*, 2015). Then, these counts were normalized for library size and transcript length using the RPKM method (reads per kilobase of transcript per million mapped reads) to normalize for sequencing depth and gene length. RPKM values for Ensembl gene identifiers corresponding to the same gene symbol were averaged, and the values associated with each gene symbol were averaged across replicates.

### Functional enrichment analysis of differently expressed mRNAs

Functional enrichment analysis was performed with *Enrichr* at https://maayanlab.cloud/Enrichr/ (Chen *et al.*, 2013). Apart from the Gene Ontology (GO) of biological processes (BP) and cellular components (CC), we selected mammalian phenotype (MP) ontology, which builds on the Mouse Genome Informatics database and is, therefore, well-suited to examine genes relevant to the mouse and its developmental biology. Terms with a FDR≤0.01 were considered enriched.

### Detection of *de novo* protein synthesis

O-propargyl-puromycin (OPP) was added to the culture medium KSOM(aa) at a final concentration of 20 µM, and the embryos were cultured for 3 h. For inhibition of protein synthesis (negative control), embryos were precultured in 50 µg/ml cycloheximide, as described (Israel *et al.*, 2019b), before switching to KSOM(aa) with OPP. The incorporation OPP was revealed using a Click-iT™ OPP Alexa Fluor™ 647 imaging kit (CAT No. C10458, Invitrogen), according to the manufacturer’s protocol. Following this procedure, all embryos were fixed with 3.7% formaldehyde for 15 min, followed by a 0.5% Triton X-100 permeabilization step for 15 min at room temperature and then incubated with the Click-iT reaction cocktail for 30 min, protected from light. Images were taken with an Andor Dragonfly spinning disc confocal unit Scanning System as described.

### Statistical analysis of developmental rates, images, and RNA sequencing results

Developmental rates of embryos and image intensities of immunofluorescence were analyzed non-parametrically by Wilcoxon test using the statistical program JMP Pro v.16 (JMP Austria, Germany & Switzerland, Heidelberg, Germany). RNA sequencing (RNAseq) data analysis was performed in-house using the output of the NextSeq^®^ 2000 System, exported in Microsoft Excel (Microsoft Deutschland GmbH, Munich, Germany) format and imported in JMP Pro v.16. Differently expressed transcripts were identified using Student’s *t*-test and the resulting *P*-values were corrected utilizing Benjamini–Hochberg’s method (FDR<0.05).

### Ethics declaration for animal experiments

Mice were used for experiments according to the license issued by the Landesamt für Natur, Umwelt und Verbraucherschutz of the State of North Rhine-Westphalia, Germany (license number LANUV 81-02.04.2017.A432; license number 81-02.04.2020.A405), in accordance with the procedures laid down in the European Directive 2010/63/EU. We observed the ARRIVE guidelines (Percie du Sert *et al.*, 2020) to the extent applicable, since the mice of this study were mostly used as oocyte donors rather than as subjects of *in vivo* experiments.

## Results

### ZP proteins are abundantly present in the place where they should no longer be—inside mouse embryos rather than outside

**Figure 2.**
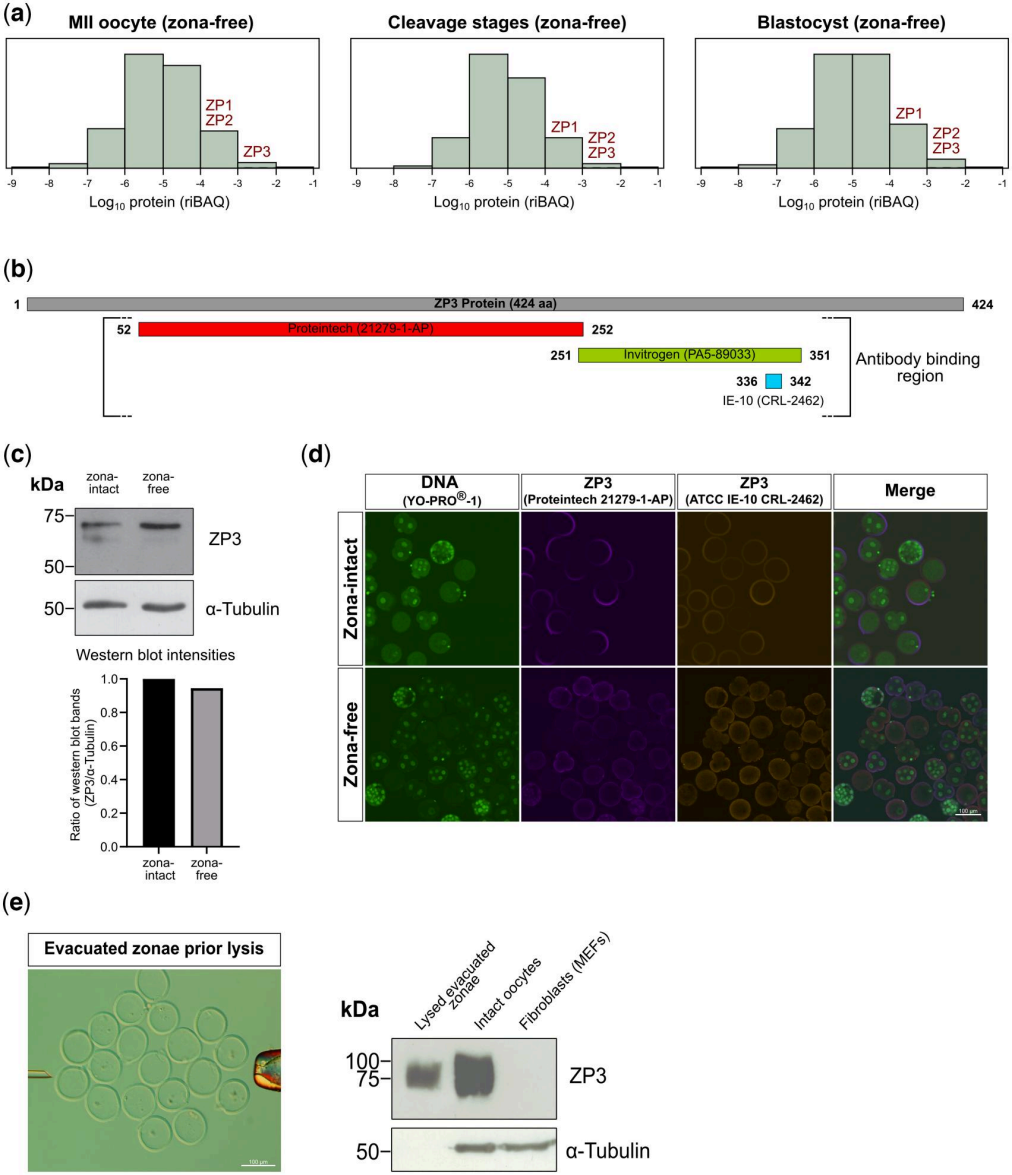
**Quantitative and qualitative expression of ZP1, ZP2, and ZP3 during mouse preimplantation development.** (**a**) Genome-wide distribution of protein abundances (riBAQ) in zona-free oocytes, cleavage stages, and blastocysts, based on the mass spectrometry dataset PXD012613 (Taher *et al.*, 2021). The proteins ZP1, ZP2, and ZP3 populate, respectively, the 82nd, 93rd, and 94th percentile of the proteome distribution of oocytes, and the 80th, 84th, and 84th percentile of the proteome distribution of blastocysts. (**b**) Three non-redundant antibodies were used to confirm the presence of ZP proteins in the inside of zona-free oocytes and embryos. Shown are the amino acid regions where the antibodies bind to ZP3. (**c**) Immunoblot analysis of ZP3 was performed on zona-intact blastocysts (n = 400) and zona-free blastocysts (n=544) as well as total ES cell lysate (30 μg) as positive control (Supplementary Fig. S2), using the antibody Invitrogen PA5-89033. The same blot was processed for ZP3, stripped, and reprocessed for α-tubulin as the loading control. Histogram shows the quantified ZP3 bands signals, normalized (zona-intact blastocysts set to 1). (**d**) Co-immunofluorescence was applied on zona-intact and zona-free embryos of mixed preimplantation stages, using the other two antibodies applied simultaneously (Proteintech 21279-1-AP; ATCC IE-10 CRL-2462). Nuclei (DNA) were stained with YO-PRO-1 and are green fluorescent. The fluorescent signal of the ZP3 protein is not uniformly distributed, neither in the extracellular ring nor in the intracellular fraction. (**e**) Immunoblotting was conducted on lysates of evacuated zonae (n=260), intact oocytes (n=260), and fibroblasts (MEFs) to demonstrate that the antibody Proteintech 21279-1-AP specifically recognized ZP3. Full-size blots of (**c**) and (**e**) are shown in Supplementary Fig. S2. riBAQ, relative intensity-based absolute quantification (Shin *et al.*, 2013), MII, metaphase II; ZP, zona pellucida.

According to longstanding notion, the zona exclusively serves its functions outside the oocyte (Fig. 1). We were therefore surprised to invariably find, in our previously generated mass spectrometric datasets (Wang *et al.*, 2016; Israel *et al.*, 2019a,b, 2021; Taher *et al.*, 2021), substantial levels of the proteins ZP1, ZP2, and ZP3 in samples of preimplantation mouse embryos although these had been stripped of their zonae prior to sample preparation (Supplementary Fig. S1). These levels were among the upper percentiles of the proteome abundance distribution (Taher *et al.*, 2021) calculated using the ‘intensity-based absolute quantification’ algorithm (Shin *et al.*, 2013), which approximates the molar fraction of the proteins (Fig. 2a).

The unexpected finding of intracellular ZP3 protein abundantly present throughout mouse preimplantation development was validated with immunoblotting and immunofluorescence using commercially available anti-ZP3 antibodies. A panel of non-redundant anti-ZP3 antibodies (binding to different regions of the ZP3 protein) was screened, of which rat monoclonal ATCC IE-10 CRL-2462, rabbit polyclonal Proteintech 21279-1-AP, and rabbit polyclonal Invitrogen PA5-89033 (East *et al.*, 1985; Grosbois *et al.*, 2019; Taher *et al.*, 2021; Yang *et al.*, 2021) (Fig. 2b) were found to work better than others, recognizing a prominent band with ancillary bands of larger size in immunoblots of blastocyst lysates. The ancillary bands are expected given the signal peptide and the glycosylation of ZP proteins, as noted in the ‘Introduction’ section. We will be using all three antibodies interchangeably, although the rabbit antibodies will be preferred over the rat antibody in *Trim-away* experiments owing to the higher affinity of TRIM21 for rabbit antibodies (Ramos *et al.*, 2022).

**Figure 3.**
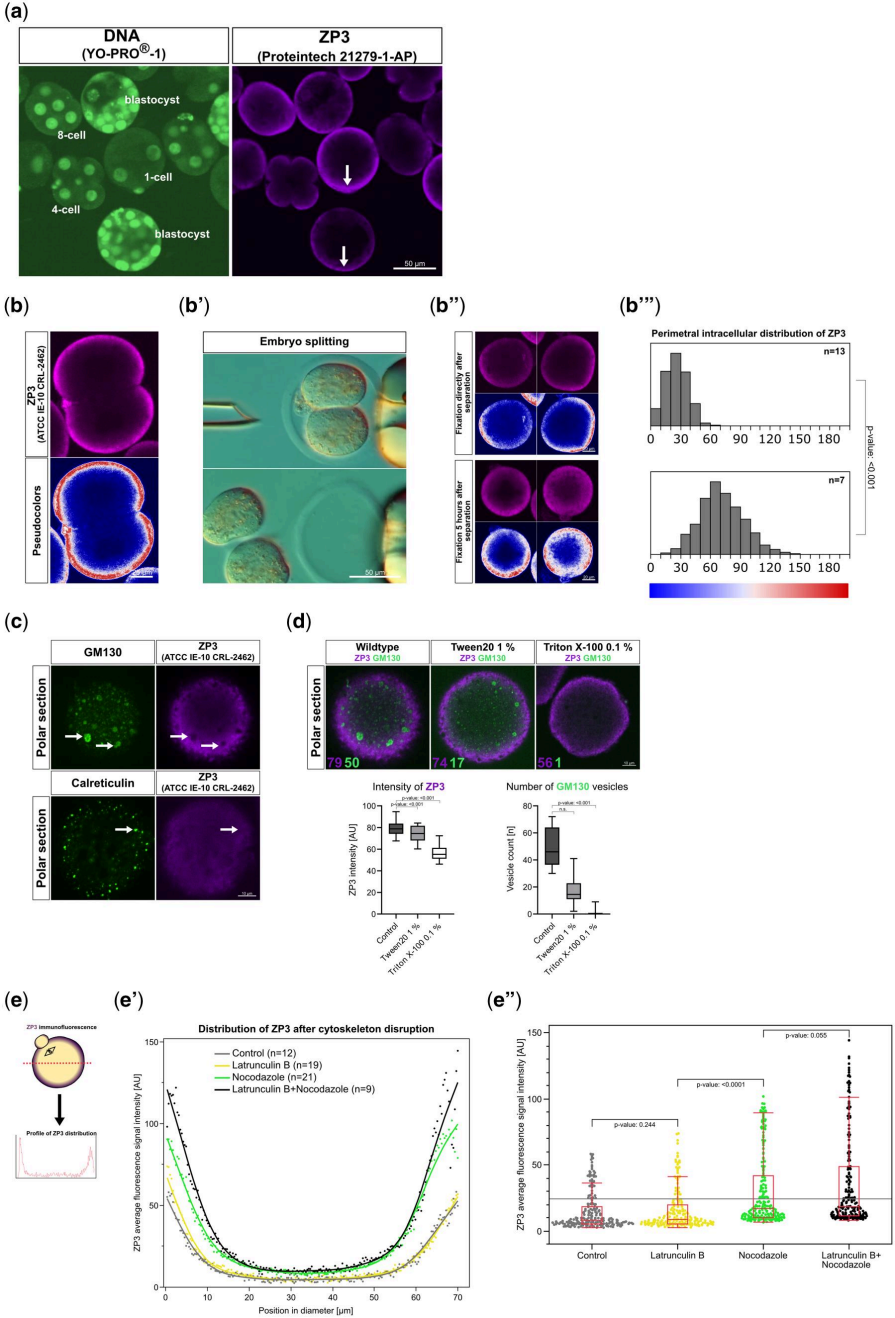
**Subcellular localization and mobility of ZP3 in mouse oocytes and embryos.** (**a**) Immunofluorescence of ZP3 in zona-free embryos. Arrows point at the non-uniform intensity of the signal located subcortically in the zygote (one-cell) and peripherally in the blastocyst (putative trophectoderm). (**b**) Peripheral intracellular location of immunostained ZP3 in two-cell stage, distributed in the shape of an arc while being excluded from the region of cell–cell contact (red, antibody; ‘union jack’ color palette, pseudocoloring). Upon mechanical blastomere separation (splitting, **b′**), the ZP3 exclusion area (pseudocolored in blue) is initially transmitted to the blastomeres but then it shrinks over time, while the ZP3 (pseudocolored in red) simultaneously expands along the perimeter (**b″**). This expansion is reflected in the shift of immunofluorescence intensities in the histograms (**b‴**), from lower values (larger blue area) to higher values (larger red area). (**c**) The immunofluorescent ZP3 signal is excluded from the areas of the Golgi and ER membranes (white arrows) as shown in optical sections taken at the polar region of oocytes after co-immunofluorescence of ZP3 (ATCC IE-10 CRL-2462) and Golgi marker GM130 and ER marker Calreticulin. (**d**) ZP3 retains 70% of its signal intensity after extraction with Triton X-100, whereas the Golgi protein GM130 is dramatically reduced (average number of vesicles reduced from 50 to 1). *P*-values calculated with Wilcoxon test. The reduction is less pronounced after extraction with the detergent Tween 20. Purple numbers indicate the ZP3 immunofluorescence intensity. Green numbers indicate the number of vesicles. (**e**) Density plot of immunofluorescent ZP3 signal quantified along the oocyte diameter after cytoskeletal disruption operated by various treatments (nocodazole and latrunculin B, individually or in combination), and statistical distribution of the immunofluorescence intensities. The curves shown in (**e′**) are averages from n oocytes (n reported next to treatment). Curves were converted to dot plots (**e″**) for statistical comparison. *P*-values calculated with Wilcoxon test. AU, arbitrary units; ZP, zona pellucida; ER, endoplasmic reticulum.

In order to validate the strong mass spectrometry signal of ZP3 by an independent method, we applied immunoblotting and immunofluorescence, thereby also addressing the subcellular localization of ZP3. Immunoblotting with antibody Invitrogen PA5-89033 returned a band of similar intensity regardless of whether the blastocysts were lysed with or without the zona (Fig. 2c and Supplementary Fig. S2a), suggesting that there is a larger amount of ZP3 present inside the blastocysts than outside them. Co-immunofluorescence with antibodies Proteintech and ATCC stained a ring around the intact embryos, consistent with common knowledge of the zona as an extracellular coat (Fig. 2d). As already seen in immunoblotting, the embryos that had been stripped of their zona (zona-free) were positive, and this was also seen for ZP1 and ZP2 (Supplementary Fig. S3). Before delving into a more detailed examination of the images, we confirmed that the ZP3 antibody signal was genuine and specific. In immunofluorescence, the signal disappeared when omitting the primary antibody (Supplementary Fig. S4). In immunoblotting, a single band of apparent molecular weight 75 kDa was produced by evacuated zonae; this band had an intensity about half that of a number of intact oocytes equal to the evacuated zonae, whereas no band was produced by fibroblasts (Fig. 2e and Supplementary Fig. S2b). *In situ*, the intracellular fraction of ZP3 was located peripherally (e.g. in the subcortical region of the one-cell stage and in the outer layer—putative trophectoderm—of the blastocyst stage), was excluded from regions of cell–cell contact in the cleavage-stage embryo, and the peripheral signal was not of uniform intensity but was stronger in some sectors (Fig. 3a and b). When cell–cell contact at the two-cell stage was loosened by incubation in Ca(2+)-free medium for 10 min followed by mechanical removal of the blastomeres from the zona (Fig. 3b′), as per our established method (Casser *et al.*, 2017), the distribution of ZP3 changed and by 5-h post-removal it had embraced almost the entire perimeter of the isolated blastomeres (Fig. 3b″ and b‴). To localize the ZP3 signal in terms of subcellular structures, we performed co-immunostaining using protein markers of the endoplasmic reticulum (Calreticulin) and Golgi complex (GM130) (Payne and Schatten, 2003). The co-immunostaining revealed that ZP3 was not associated with these endomembrane systems (Fig. 3c). To figure out where else ZP3 may be located if not bound to membranes, we applied the method of live-oocyte extraction using the detergent Triton X-100: this allows to distinguish between ‘soluble’ or ‘solubilizable’ proteins and proteins that are bound in fibrillar sheets (Gallicano *et al.*, 1994; Kim *et al.*, 2010). While GM130 was extracted by Triton X-100, ZP3 was refractory to the harsh procedure and more than half of the ZP3 amount was still present in oocytes after the extraction (Fig. 3d). Although ZP3 withstood the extraction with Triton X-100, it did not withstand drugs that disrupt the cytoskeleton. Polymerization inhibitors latrunculin B (actin) and nocodazole (tubulin) were applied to oocytes for 5 h. We then checked whether the intracellular ZP3 was still localized in the subcortical region or had dispersed away from there. We observed that ZP3 dispersed from the otherwise compact cortical deposit after treatment with either nocodazole alone or nocodazole+latrunculin B, but not after treatment with latrunculin B or with solvent alone, as revealed by density plots taken across randomly chosen oocyte diameters after immunofluorescence (Fig. 3e, e′ and e″).

Together, these findings—enabled by three different methods and distinct antibodies—discount the possibility that the detection of ZP proteins inside the embryos is a false positive. The cytoskeleton disruption experiments reveal a new dimension in the cellular localization of ZP3 protein, which is associated not only with secretory membranes, but also with the microtubules of the cytoskeleton. We hypothesized that there must be a hitherto overlooked intracellular fraction of ZP proteins that persist until the blastocyst stage in a non-membrane-associated form. Therefore, we became interested in the origin, mechanistic basis, and functional meaning of this unusual fraction of ZP proteins found inside the embryos rather than outside.

### Intracellular ZP proteins found in the blastocyst are still those synthesized during oogenesis

**Figure 4.**
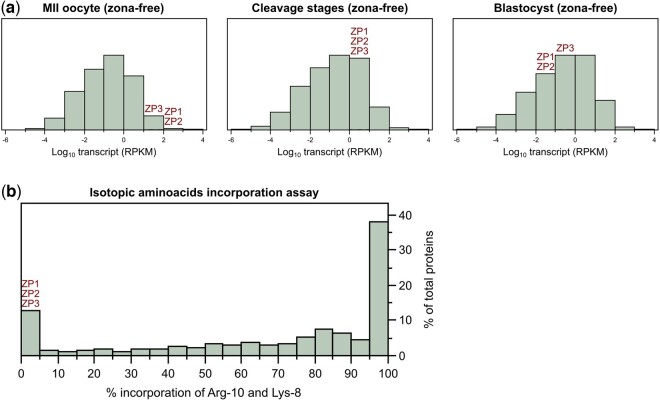
**Abundant ZP transcripts are not translated but ZP proteins found in mouse embryos are of maternal origin.** (**a**) Genome-wide distribution of transcript abundances (RPKM) in oocytes, cleavage stages, and blastocysts, based on the dataset deposited in the DNA Databank of Japan Sequence Read Archive with the dataset identifier DRA005956 and DRA006335 (Taher *et al.*, 2021). The mRNAs *ZP1*, *ZP2*, and *ZP3* populate, respectively, the 97th, 95th, and 97th percentile of the transcriptome distribution of oocytes, and the 41st, 46th, and 57th percentile of the transcriptome distribution of blastocysts. (**b**) When zygotes are cultured 4 days in the presence of Arg-10 and Lys-8, both amino acids were not incorporated in the ZP proteins found inside the blastocysts, unlike the majority of blastocyst proteins that are labeled by 83% on average (n=1719 proteins; dataset PXD035570). The dataset is available in PRIDE repository with accession number PXD035570 and is provided in simplified form in Supplementary Table S1. RPKM, reads per kilobase of transcript per million mapped reads; ZP, zona pellucida.

Two non-mutually exclusive possibilities are that the intracellular fraction of ZP proteins is a leftover from oogenesis, or that it is produced new in the embryos: the former possibility is based on the principle of parsimony, the latter on the fact that the *ZP* genes’ transcripts are present in embryos, as we show hereafter, and that neozona formation was hypothesized to occur in embryos, particularly of rabbits (Denker, 2000; Menkhorst and Selwood, 2008). It should be noted, however, that the exact origin and molecular composition of the hypothetical neozona is still unclear. To distinguish between the possibilities, we compared the synthesis of ZP proteins against the abundance of *ZP* transcripts. Although the transcription of *ZP* genes is oocyte-specific and >95% of *ZP3* mRNA is reportedly degraded during ovulation (Roller *et al.*, 1989; Liang *et al.*, 1990), reanalysis of our previously generated RNAseq data (Taher *et al.*, 2021) revealed that the *ZP* transcripts are in fact not as scarce in embryos as previously assumed. At the blastocyst stage, for example, the *ZP* transcripts populated the median region of the transcriptome abundance distribution (Fig. 4a). Intriguingly, the *ZP* transcripts also featured isoforms (Supplementary Fig. S5a). The majority (>90%) of ZP3 transcripts mapped to the isoform ‘201’ (Ensembl annotation) that encodes the canonical ZP3 protein with 424 amino acids (UniProt P10761); the residual fraction of ZP3 transcripts mapped to the isoform ‘202’ (Ensembl annotation) that encodes a shorter protein of 136 amino acids featuring an incomplete signal peptide at the N-terminus and lacking almost 300 amino acids at the C-terminus (Uniprot F6VD35) (Supplementary Fig. S5b).

To resolve if these transcripts are simply present or are also translated, and if they produce two protein variants of ZP3, we labeled the proteins that are synthesized in zona-intact zygotes by feeding these with the non-radioactive isotopic amino acids Arg-10 (^13^C_6_$H_{14}^{15}$N_4_O_2_) and Lys-8 (^13^C_6_$H_{14}^{15}$N_2_O_2_) dissolved in the culture medium. Cells cannot distinguish these isotopic amino acids from the natural counterparts, because there is no difference apart from the atomic weight of Carbon and Nitrogen. As a result, Arg-10 and Lys-8 are taken up and used in an entirely physiological way. Indeed, labeling did not perturb the normal developmental schedule: the zygotes formed blastocysts on Day 4 (91%±3%, seven replicates) at a rate similar to control embryos cultured in conventional medium (81%±13%, 13 replicates), and the labeled blastocysts also developed to term when transferred surgically to pseudopregnant uteri (216 blastocysts transferred to 27 females, resulting in 14 pregnancies, 59 implantations, 39 fetuses at term). With the safety of this isotopic labeling that preserved developmental potential, we subjected the labeled blastocysts to zona removal followed by proteome analysis. Samples of ∼500 zona-free blastocysts were collected and subjected to mass spectrometry. In total, 1719 proteins were detected and quantified. A median labeling rate of 83% resulted in 1515 labeled proteins, and the remaining 204 proteins including ZP1, ZP2, and ZP3 were detected as not labeled (Fig. 4b and Supplementary Table S1). Analysis of our mass spectrometry data could not resolve the short from the long isoform of ZP3, because the tryptic fragments mapped to a region that is common to the two isoforms or to a region that is exclusive to the long isoform but not to the short isoform (Supplementary Fig. S5b). A provisional analysis of the peptide fragment intensities suggested, however, that the bulk of ZP3 protein was the canonical long isoform, given that the intensity per peptide was not significantly higher where the two isoforms overlapped than where only the long isoform was represented (Supplementary Fig. S5c). We next characterized features that distinguish non-labeled proteins, subjecting them to gene and MP ontology analysis using *Enrichr* (Chen *et al.*, 2013). The most prominent terms were ‘mitochondrial matrix’ in the cellular component (GO CC:0005759), ‘cellular amino acid catabolic process’ in the biological process (GO BP:0009063), and ‘abnormal oocyte morphology’ in the mammalian phenotype (MP:0001125). The last term is consistent with the occurrence—among the 204 proteins—of oocyte-specific and maternal-effect genes (e.g. *Nlrp2, Nlrp5, Nlrp9, Npm2, Padi6, Tle6, Uhrf1* (Mitchell, 2022); we also note their known involvement in mitochondrial function (Fernandes *et al.*, 2012)).

Taken together, these results reveal that although the active transcription of ZP genes terminates with the end of oogenesis, the protein products persist during the entire preimplantation phase. Although commonly thought of as a single protein, ZP3 features in reality two variants, of which the form already known to all (the long isoform) is prevalent. This finding of oocytic proteins present within the cytoplasm of embryos elicits considerations about the definition of ‘oocyte-specific’ genes and begs the question as to why embryos hold inside them large amounts of a protein that widespread consensus ascribe with extracellular functions around oocytes.

### An inside job for ZP3: functional inhibition or physical degradation (knockdown) of ZP3 protein hampered the first zygotic cleavage

**Figure 5.**
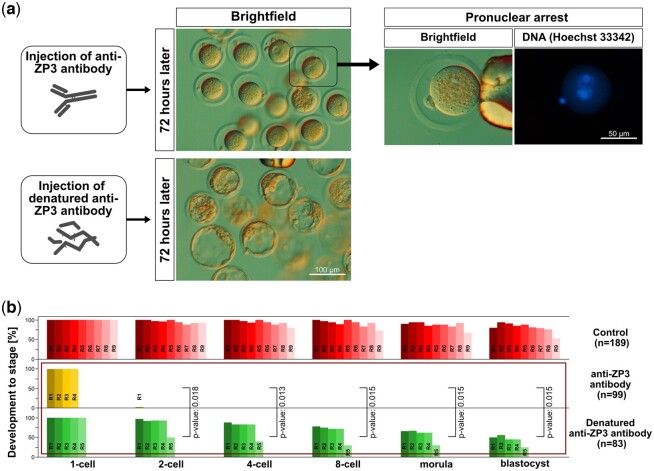
**Epitope masking of ZP3 by antibody hampers the first zygotic cleavage in mice.** (**a**) Representative images of zygotes that remained arrested at the pronuclear stage when microinjected with anti-ZP3 (Proteintech 21279-1-AP), in contrast to the blastocyst progression of zygotes microinjected with the heat-denatured antibody. Pronuclear-stage arrest was confirmed at higher magnification following Hoechst 33342 staining for DNA. Note the two stained pronuclei. (**b**) Developmental rates of the zygotes shown in (**a**), compared to control zygotes (untreated, no microinjection). In each of the three groups, the multiple bars next to each other stand for the replicates (anti-ZP3, four replicates; denatured anti-ZP3, five replicates; non-manipulated, i.e. intact, nine replicates), and the rates of development were normalized to the one-cell stage (100%). For the replicate of the same group, the color is the same, but in different shades. Square brackets between the two series of anti-ZP3 and denatured anti-ZP3 indicate the statistical comparison and its *P*-value (Wilcoxon test). The complete dataset and its breakdown into replicates are provided in Table 1. ZP, zona pellucida.

Given the observation that *ZP* mRNAs are present but not translated in embryos (Fig. 4b), it would be futile to test for the developmental relevance of the intracellular ZP proteins by disrupting the DNA loci or depleting the mRNAs. Therefore, we tackled the intracellular fraction directly at the protein level in zona-intact embryos. Given the refractoriness of ZP3 to extraction with Triton X-100 (Fig. 3d), we reasoned that ZP3 should be outside vesicles and thus accessible to antibodies. We followed a dual approach in which ZP3 was inhibited (epitope masking) versus depleted in amount (knockdown). As pharmacological inhibitors do not exist for ZP3, we microinjected zygotes with defined amounts of either of two anti-ZP3 antibodies, Invitrogen PA5-89033 and Proteintech 21279-1-AP, in purified form (see Materials and methods). Most of the zygotes failed to reach the blastocyst stage when microinjected with Invitrogen PA5-89033 and they even failed to cleave a single time when microinjected with Proteintech 21279-1-AP, being arrested at the pronuclear stage (Table 1; Fig. 5a and b; *P* = 0.018; Wilcoxon test). Both pronuclei had migrated to the middle of zygotes (suggestive of normal actin function) and pronucleus-arrested zygotes remained as single cells for 4 days, without fragmenting or degenerating (Fig. 5a). Developmental arrest was associated with the antibody-epitope binding, since blastocyst formation was not impacted when the antibody was heat-denatured prior to microinjection (Fig. 5a and b). These results lend support to our proposal that ZP3 is needed for hitherto overlooked embryonic processes.

**Table 1.** Fertilized mouse oocytes (zygotes) used in the epitope masking and knockdown (Trim-away) experiments.

	Replicates	Zygotes used	Microinjection survival rate	Normalized developmental rates %±SD					
Microinjection*	N	N	%±SD	One-cell	Two-cell	Four-cell	Eight-cell	Morula	Blastocyst
none (not manipulated)	9	189	n.a.	100	96 ± 4	94 ± 7	91 ± 9	87 ± 9	81 ± 12
*mCherry-Trim21* mRNA	6	195	61 ± 13	100	96 ± 5	86 ± 6	79 ± 9	72 ± 13	65 ± 13
anti-ZP3**	4	143	69 ± 15	100	1 ± 1	–	–	–	–
anti-ZP3***	1	39	47	100	97	90	90	90	41
denatured anti-ZP3**	5	146	57 ± 12	100	85 ± 20	75 ± 20	65 ± 20	57 ± 15	44 ± 12
denatured anti-ZP3***	2	85	63 ± 4	100	86 ± 10	79 ± 10	75 ± 6	75 ± 6	62 ± 20
Trim-away ZP3**	8	577	60 ± 17	100	16 ± 34	12 ± 33	5 ± 14	2 ± 6	–
Trim-away ZP3***	3	109	55 ± 14	100	83 ± 12	71 ± 02	54 ± 18	45 ± 24	27 ± 17
buffer of anti-ZP3*+*mCherry-Trim21* mRNA	5	149	62 ± 14	100	90 ± 10	84 ± 12	76 ± 12	68 ± 14	53 ± 17
Trim-away ZP1	2	62	73 ± 16	100	61 ± 0	51 ± 0	49 ± 0	32 ± 0	29 ± 20
Trim-away ZP2	3	82	68 ± 23	100	90 ± 10	21 ± 18	2 ± 3	–	–

The table shows the total number of replicates and zygotes manipulated in this study. The developmental rates of the pooled replicates are normalized to the one-cell stage (zygote). Oregon Green dextran beads were used as a tracer in all experiments, to confirm that the microinjection had succeeded technically.

* Oregon Green dextran beads are always present as a microinjection tracer.

** Proteintech antibody, 21279-1-AP.

*** Invitrogen antibody, PA5-89033.

ZP, zona pellucida.

Since the epitope masking of ZP3 by the antibody was sufficient to cause one-cell arrest, we reasoned that ZP3 must support critical cytoplasmic processes. We wondered, therefore, whether the effect would turn out to be even more pronounced when ZP3 is not only masked, but also degraded. To this end, we adopted the method of TRIM21-mediated proteasomal degradation, briefly known as ‘*Trim-away*’ (Clift *et al.*, 2017), which we previously modified for better applicability to large amounts of substrate, such as those characteristically accumulated in oocytes (Israel *et al.*, 2019a). The ‘*Trim-away*’ of ZP3 was achieved by microinjection of the same antibodies previously used (either Invitrogen PA5-89033 or Proteintech 21279-1-AP), together with the mRNA of the ubiquitin ligase TRIM21 (*mCherry-Trim21* mRNA), thereby triggering the proteasomal commitment of the ternary complex formed between target protein, antibody, and mCHERRY-TRIM21. The effect of Proteintech was stronger (Table 1) and this antibody was used preferentially in the subsequent experiments. It may be noted that the antibody Proteintech 21279-1-AP binds in a region that is spanning the canonical and the short isoforms of ZP3 (Fig. 2b).

**Figure 6.**
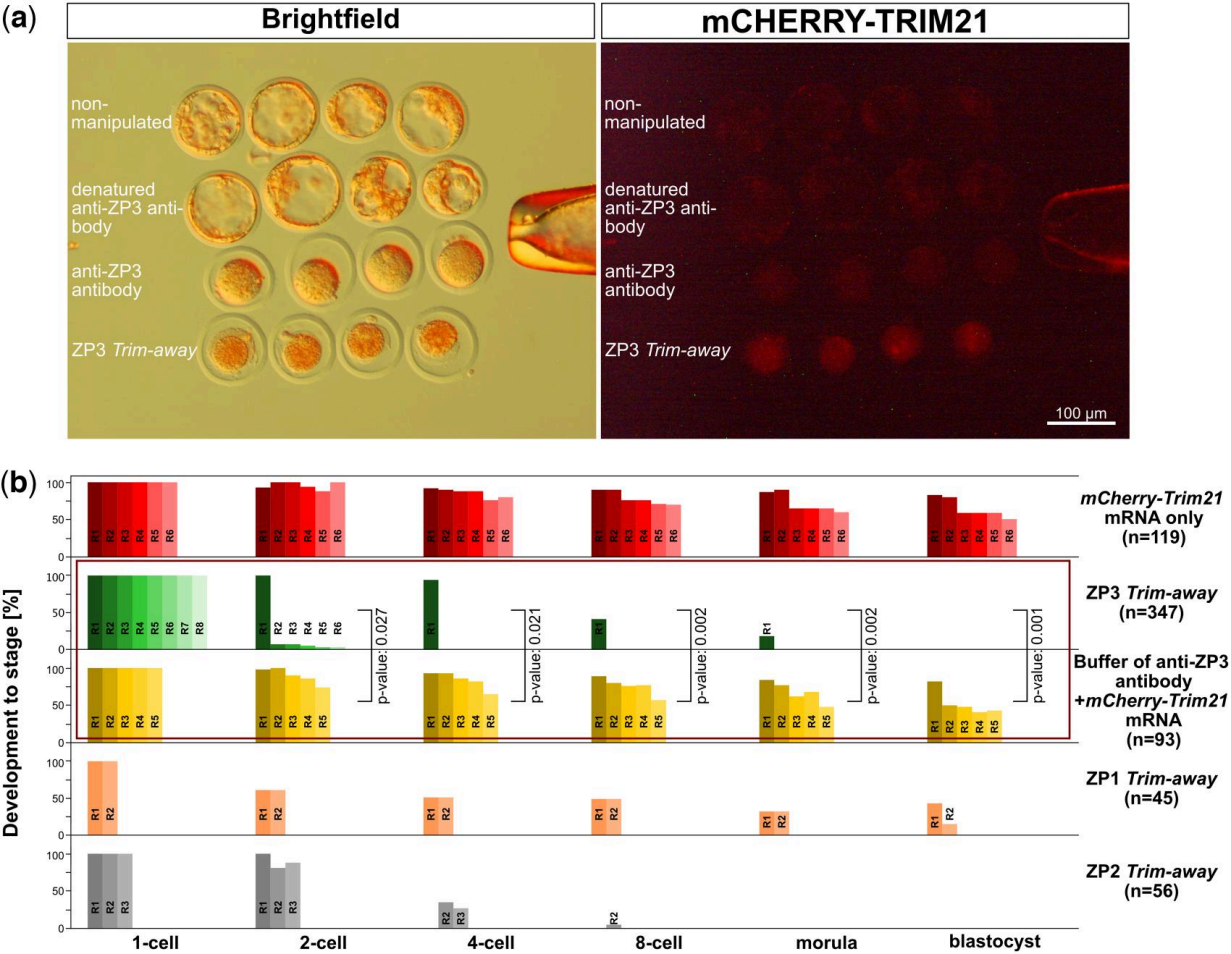
**Degradation of ZP3 in mouse zygotes via *Trim-away* exacerbates the effect of epitope masking by antibody alone.** (**a**) Representative images of zygotes that not only fail to cleave but also degenerate when anti-ZP3 antibody (Proteintech 21279-1-AP) is coinjected with *mCherry-Trim21* mRNA (*Trim-away*). mCHERRY is the fluorescent tag of TRIM21. (**b**) Developmental rates of the zygotes subjected to *Trim-away* or its controls, e.g. microinjection of *mCherry-Trim21* mRNA only or mRNA with antibody buffer (third flow-through of the Amicon filter device). In each of the five series, the multiple bars next to each other stand for the replicates (*mCherry-Trim21* mRNA only, six replicates; ZP3 *Trim-away*, eight replicates; antibody buffer+*mCherry-Trim21* mRNA, five replicates; ZP1 *Trim-away*, two replicates; ZP2 *Trim-away*, three replicates) and the rates of development were normalized to the one-cell stage (100%). For the replicates of the same group, the color is the same, but in different shades. *Trim-away* of ZP3 hampered the first cleavage, *Trim-away* of ZP2 hampered the second cleavage, while *Trim-away* of ZP1 allowed for blastocyst formation but the blastocysts were consistently smaller and less robust than controls. The complete dataset and its breakdown into replicates are provided in Table 1. Square brackets between the two series of ZP3 *Trim-away* and antibody buffer indicate the statistical comparison and its *P*-value (Wilcoxon test). ZP, zona pellucida; TRIM21, tripartite motif containing 21.

In our routine setting, the reagents are co-injected as a cocktail (Supplementary Fig. S6a), which has the advantage to inflict a single microinjection on the zygote. However, this advantage is traded off against the viewing of the consecutive rise and fall of mCHERRY-TRIM21 fluorescence, because mCHERRY-TRIM21 starts to be degraded as soon as the *mCherry-Trim21* mRNA has been translated in sufficient amounts. Therefore, we injected the two reagents separately for the sole purpose of a proof-of-principle visualization: *mCherry-Trim21* mRNA in the zygote, first, followed by ZP3 antibody in one of the two blastomeres originated from the first zygotic cleavage (Supplementary Fig. S6b). This allowed us to compare the intensity of mCHERRY-TRIM21 fluorescence in the *Trim-away* blastomere against the other blastomere that contained the same initial amount of mCHERRY-TRIM21: *Trim-away* caused a decline of mCHERRY signal (Supplementary Fig. S6b), which was seen also at the ZP3 protein level (Supplementary Fig. S6c). With the comfort of this proof, we went back to the routine microinjection of the cocktail in the zygote. The biological effect was more severe compared to the microinjection of the sole Proteintech antibody without TRIM21 (Fig. 6a and b; Table 1; *P* ≤ 0.027; Wilcoxon test): zygotes subjected to *Trim-away* were not only pronucleus-arrested, but also degenerated after 2 days, unlike the zygotes that remained single-celled but alive when receiving the anti-ZP3 antibody alone (Fig. 6a). In contrast to the knockdown, the overexpression of ZP3 by way of *ZP3-eGFP* mRNA microinjection in zygotes was permissive of blastocyst formation (62%±15% of one-cell embryos, N = 135). Moreover, in this experiment, we saw a subcellular localization of ZP3-eGFP consistent with that of the endoplasmic reticulum.

Despite the lethal effect of ZP3 *Trim-away*, the degradation of ZP3 was only partial, i.e. a knockdown, as revealed by staining of the residual ZP3 with antibody ATCC IE-10 CRL-2462 (Supplementary Fig. S6c; Supplementary Table S4). Clearly, if such a harsh chemical means as Triton X-100 could not remove all of the ZP3 (Fig. 3d), then neither could *Trim-away*. We recalled that nocodazole had succeeded in dispersing ZP3 away from its subcortical localization (Fig. 3e, e′, and e″). Therefore, we wondered if treatment with nocodazole during *Trim-away* would make ZP3 more accessible and thereby enhance the depletion. The zygotes were subjected to microinjection of *Trim-away* reagents first, followed by treatment with nocodazole and latrunculin B, without, however, providing evidence of enhanced ZP3 depletion (Supplementary Fig. S7).

The pronuclear arrest observed so far was based on the targeting of ZP3 in zygotes. We therefore asked if our *Trim-away* findings were true also when the oocyte has yet to be activated, and if similar results would manifest also after targeting of ZP1 and ZP2. We subjected MII oocytes to ‘*Trim-away*’ of ZP3 before activating with Strontium chloride (parthenogenesis). All treated and activated oocytes remained arrested at the one-cell stage (N = 0 two-cell embryos/26 oocytes; Proteintech 21279-1-AP). We also applied *Trim-away* to ZP1 and ZP2 (Supplementary Fig. S8). After 4 days, zygotes microinjected with *mCherry-Trim21* mRNA together with anti-ZP2 antibody were found arrested at the two- and four-cell stage (0 blastocysts/56 zygotes; Table 1; Fig. 6b; Supplementary Fig. S8a). Zygotes receiving anti-ZP1 antibody progressed to blastocysts but these were stunted, i.e. smaller and less expanded than those from zygotes receiving the sole *mCherry-Trim21* mRNA (18 blastocysts/45 zygotes; Table 1; Fig. 6b; Supplementary Fig. S8b). As in the case of ZP3, these defects were not caused by the microinjection *per se*: zygotes that received the antibody buffer, or the heat-denatured anti-ZP1 or anti-ZP2 antibody, progressed efficiently to blastocysts (Table 1).

Taken together, these results reveal that mouse embryos have a hitherto overlooked internal requirement for ZP proteins (Fig. 1). The ZP proteins found inside are not a ballast left from oogenesis, but serve an active role during mouse preimplantation development. Among the three ZP proteins, the requirement of ZP3 is the earliest and strongest, followed by that of ZP2 and then ZP1, in a chronological precession and decreasing severity of phenotype (ZP3 > ZP2 > ZP1). We focused our further molecular investigation on the earliest and boldest of the three phenotypes, namely that of ZP3.

### Knockdown of ZP3 interferes with cytoplasmic translation in the context of EGA

Characterizing the transcriptome is a common way to understand what specific genes are required for. To illuminate the requirement of ZP3 for pronuclear stage progression, we examined the transcriptome at 10 and 24 h past the zygotes’ microinjection with the *Trim-away* cocktail, compared to that of controls (*mCherry-Trim21* mRNA combined with the buffer of the ZP3 antibody). We also included non-microinjected zygotes, to verify the successful supply of *mCherry-Trim21* mRNA in the other two groups.

**Figure 7.**
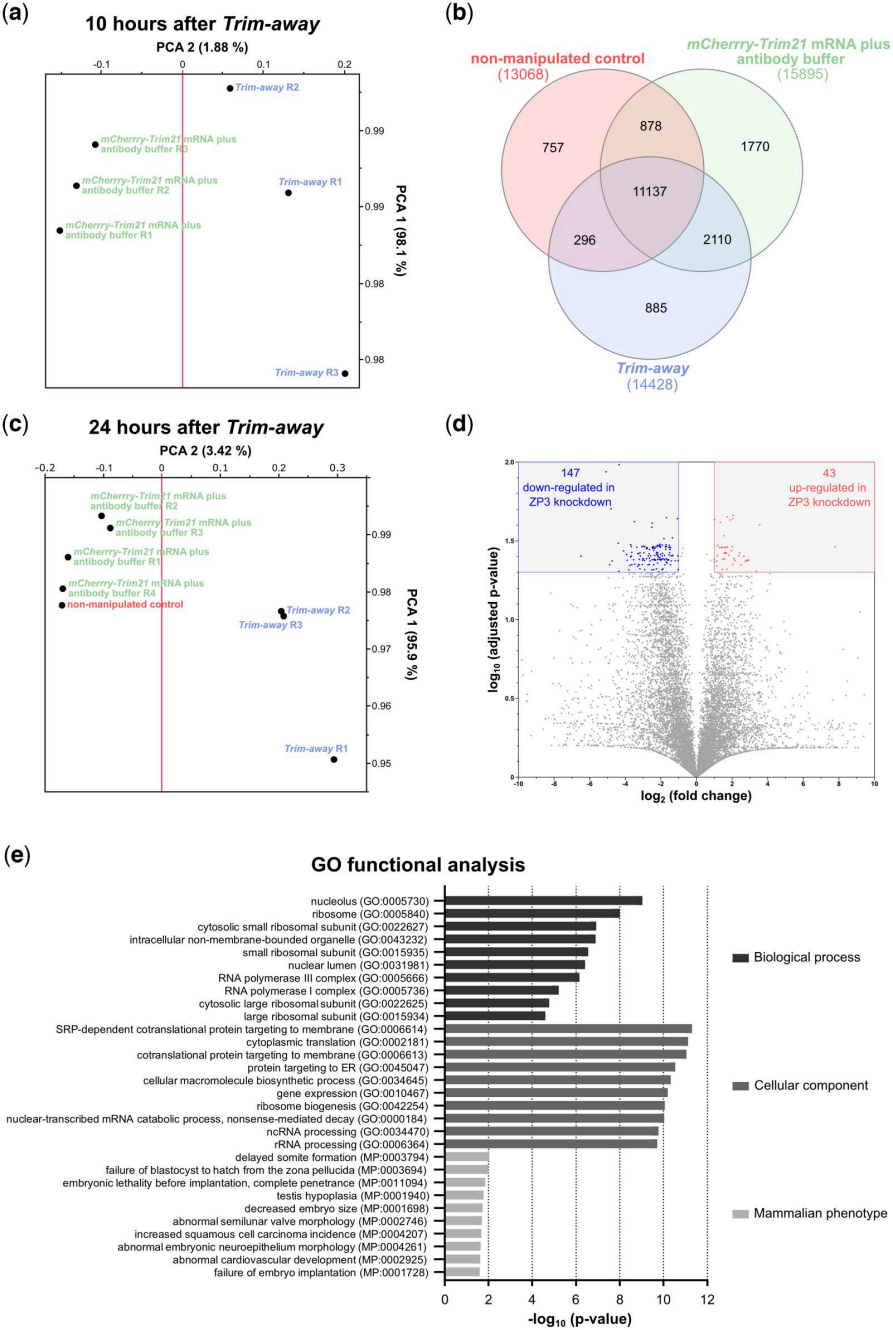
**Effects of ZP3 knockdown on the mouse embryonic transcriptome.** (**a**) Principal component analysis (PCA) of the zygotes that were subjected to transcriptome analysis 10 h after microinjection of *mCherry-Trim21* mRNA with anti-ZP3 (Proteintech 21279-1-AP) or its buffer. (**b**) At 24 h, a new dataset was generated (main) in which zygotes were subjected to transcriptome analysis same as in **(a)** except that the time between microinjection and sampling was longer. A third group of non-injected zygotes was also included. For these three conditions, the Venn diagram describes the overlap in detected transcripts at 24 h. The RNAseq datasets are available in Gene Expression Omnibus (GSE232142, 10h; GSE203626, main) and the main dataset is also provided in simplified form in Supplementary Table S2. (**c**) PCA based on the core of 11 137 transcripts that are common to all samples of the main dataset. Each point corresponds to one sample (*mCherry-Trim21* mRNA with antibody buffer in triplicate, ZP3 *Trim-away* in quadruplicate, non-manipulated in unicate). The first two principal components (PCs) of the data are represented. The PCA of the transcriptome resolves the ZP3 *Trim-away* and the other groups in the second component. (**d**) Volcano plot showing the effect of ZP3 *Trim-away* on the embryo transcriptome at the chronological two-cell stage compared to *mCherry-Trim21* mRNA plus antibody buffer (24 h dataset). The numbers of mRNAs that are under- versus over-expressed (fold change >2, adj.*P*-value < 0.05, *t*-test) are shown in the upper corners. (**e**) The 198 differently expressed mRNAs were subjected to GO analysis using *Enrichr* (Chen *et al.*, 2013), and the top-10 terms (ranked by *P*-value) were visualized for three ontologies: GO ‘biological process’, GO ‘cellular component’, and mammalian phenotype ontology. ZP, zona pellucida; GO, gene ontology.

Already, at 10 h, the ZP3 *Trim-away* embryos were distinguishable from the non *Trim-away* controls in principal component analysis (PCA), although the differences in gene expression were still on the rise and did not reach statistical significance given the short time available to *Trim-away* to mount its effect (Fig. 7a). This dataset (GSE232142) was not investigated further. The effects on gene expression became overt at 24 h. Of 17 833 gene transcripts detected in total, 11 137 were expressed in common (three samples *mCherry-Trim21* mRNA and antibody buffer, four samples ZP3 *Trim-away*, one sample non-manipulated; Fig. 7b) and were used for further analysis. The RNAseq dataset is deposited (GSE203626; Supplementary Table S2). In PCA, the ZP3 *Trim-away* embryos were distinct from those that received the antibody buffer in lieu of the ZP3 antibody in conjunction with *mCherry-Trim21* mRNA, and the latter were not distinct from the non-microinjected controls (Fig. 7c), documenting that microinjection *per se*—while being invasive—has a negligible effect on gene expression.

We focused our attention on the significant differences between the *Trim-away* embryos and the embryos that received the sole *mCherry-Trim21* mRNA. After adjusting for false discovery (*P*<0.05 after Benjamini–Hochberg correction), our analysis of the 11 137 transcripts identified 198 differently expressed transcripts between ZP3 *Trim-away* embryos and *mCherry-Trim21*-expressing controls. Of the 198 transcripts, 190 also exceeded a 2-fold change (147 down, 43 up; Volcano plot, Fig. 7d).

**Figure 8.**
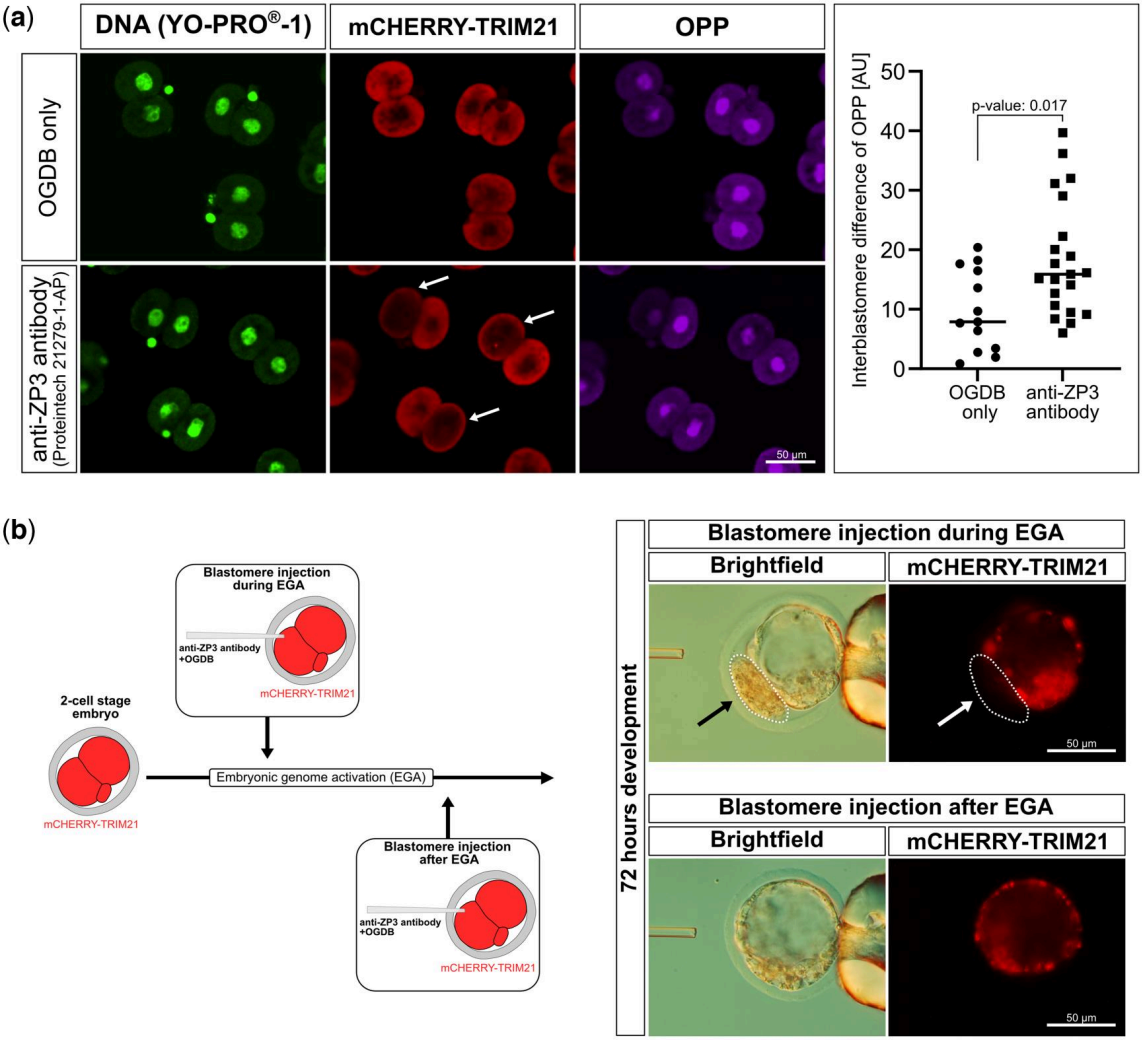
**Effects of ZP3 knockdown on cytoplasmic translation and distinct effects of the knockdown depending on the early versus late phase of the two-cell stage in mice.** (**a**) Zygotes were preloaded with *mCherry-Trim21* mRNA, and after one cleavage they were microinjected with OGDB or anti-ZP3 (Proteintech 21279-1-AP) in one blastomere only, to then be subjected to analysis of protein synthesis using Click-iT™ OPP Alexa Fluor™ 647 imaging kit. Note the reduction of mCHERRY fluorescence in the blastomere receiving anti-ZP3 (white arrows) while the blastomere receiving OGDB retained its fluorescence. OPP fluorescence intensities were measured in both blastomeres of each embryo, and used to calculate absolute interblastomere differences of OPP signal intensity in the *Trim-away* versus control (OGDB microinjection) groups (*P*=0.017; Wilcoxon test). The interblastomere difference is larger in *Trim-away* than in control, because ZP3 knockdown reduces the protein synthesis in one blastomere. The raw measurement data are provided in Supplementary Table S3. (**b**) Using the same design as in (**a**) but delaying the anti-ZP3 injection, the knockdown of ZP3 did not result in blastomere arrest when conducted at the late two-cell stage compared to the early two-cell stage. After 72 h of culture, the blastomere that underwent *Trim-away* at the early two-cell stage produced no cell progeny (arrows and dotted circle), whereas both blastomeres participated in blastocyst formation when *Trim-away* was applied at the late two-cell stage. AU, arbitrary units; EGA, embryonic genome activation; OPP, O-propargyl-puromycin, ZP, zona pellucida; OGDB, Oregon Green dextran beads.

Since the knockdown of ZP3 caused one-cell arrest, we asked how the 190 transcripts compare to the catalog of genes whose mutations produce preimplantation lethality in mice, as listed under ‘MP:0006204’ of the MP ontology. We found seven such genes among the 190 (*Cdca8, Eif6, Kpna7, Kif11, Orc6, Psmc4, Timm23*). This is not a high proportion (4%) yet it suggests that the knockdown of ZP3 compromises the embryos anyway, on multiple levels, and that the pronuclear stage is simply the time when the first process is affected. To illuminate what these processes could be, we subjected the set of 190 transcripts to gene and MP ontology analysis using *Enrichr* (Chen *et al.*, 2013). This returned top terms that relate to protein and ribosomal RNA metabolism, such as ‘SRP-dependent cotranslational protein targeting to membrane’ (GO BP:0006614), ‘cytoplasmic translation’ (GO BP:0002181), ‘nucleolus’ (GO CC:0005730), and ‘ribosome’ (GO CC:0005840) (Fig. 7e), as well as embryo survival, such as ‘failure of blastocyst to hatch from the zona pellucida’ (MP:0003694) (Fig. 7e). In regards to ‘cytoplasmic translation’, we recalled that zygotes and early embryos undergo proteome remodeling (Latham *et al.*, 1991) and that interfering with protein synthesis prevents transcriptional activation of the embryonic genome (Wang and Latham, 1997). Therefore, we measured nascent protein synthesis after incorporation of OPP, as per our established protocol (Israel *et al.*, 2021). The OPP reaction was specific, since it was abolished by preculture with cycloheximide. OPP measurements were performed in two-cell embryos preloaded with *mCherry-Trim21* mRNA at the one-cell stage and then microinjected with anti-ZP3 in one blastomere (control group received OGDB in lieu of antibody); this allowed for internal normalization of treatment and control groups. The blastomere that underwent ZP3 knockdown presented lower protein synthesis as captured by the interblastomere ratio of fluorescence intensities after the Click-chemistry reaction (Fig. 8a and Supplementary Table S3). We also recalled that embryos intensify their RNA metabolism to accompany the EGA. Therefore, we searched the 190 transcripts for terms known to be regulated during EGA (Datasets S1–S5 in Abe *et al.*, 2018), finding 159 of them. The majority of these transcripts (121/159) were mapped to clusters that are upregulated during the minor or major wave of EGA in normal mouse development, while these transcripts were reduced in the pronucleus-arrested zygotes microinjected with ZP3 *Trim-away*. By contrast, the remaining 38 transcripts mapped to the only cluster ‘consisting of the genes whose expression levels continuously decreased after fertilization and cleavage to the two-cell stage’ (Abe *et al.*, 2018), while these transcripts failed to be downregulated in zygotes microinjected with ZP3 *Trim-away* (Supplementary Fig. S9).

From the above results, it appears that zygotes with knockdown of ZP3 are not apt to manage the RNA and protein dynamics that accompany the oocyte-to-embryo transition. We reasoned that if those changes were the cause of EGA disruption, then *Trim-away* of ZP3 should not matter if conducted at a time when EGA has already occurred. To test this hypothesis, we applied *Trim-away* of ZP3 either early during the two-cell stage, when EGA is occurring, or late during the two-cell stage, when EGA has occurred, using the sister blastomeres as reciprocal controls. When *Trim-away* of ZP3 was applied in one blastomere of the early two-cell embryo, it prevented cleavage of the injected blastomere, resulting in a mini-blastocyst formed by the other (untreated) blastomere. By contrast, the blastomere progressed further and participated in blastocyst formation when the same treatment was applied at the late two-cell stage (Fig. 8b).

Collectively, our results corroborate that ZP3 is required for the oocyte-to-embryo transition in mice. The inhibitory effect on protein synthesis brought about by *Trim-away* of ZP3 is probably a direct cause of one-cell arrest (consistent with the notion that protein synthesis inhibition with, for example, cycloheximide causes one-cell arrest). Even if the zygotes made it through this hurdle, they would have a problem shortly after, when the knockdown of ZP3 hampers the major phase of EGA, exemplified by the failed downregulation of otherwise downregulated maternal transcripts, as revealed by RNAseq. However, the zygotes are expected to be on the safe side thereafter, as demonstrated by the blastocyst progression of blastomeres subjected to *Trim-away* at the late two-cell stage, when the bulk of EGA has already occurred.

## Discussion

The significance of the findings presented here is 3-fold. First, our results expose a hitherto unknown fourth function of the ZP proteins in a mouse model, in addition to the traditional functions of: encasing the oocytes in ovarian follicles; mediating sperm–oocyte interaction; and protecting the preimplantation embryo from physical injury while holding the blastomeres together: as a fourth function, there is also embryo survival and development. Second, our results document that the newly discovered function of ZP3 is operating intracellularly—an inside job for a protein hitherto believed to operate only outside the cell. This intracellular function hinges on a remarkably stable deposit of ZP3 that resists the harsh chemical extraction of membranes with the detergent Triton X-100, while it is sensitive to microtubule depolymerization. A similar behavior was previously observed for the oocytic isoform of the DNA methyltransferase 1 (Dnmt1o), which was retained following permeabilization procedures that released more than 75% of total soluble protein (Doherty *et al.*, 2002), except that Dnmt1o was not sensitive to cytoskeletal disruption while ZP3 was. Third, our results suggest that the newly discovered function of ZP3 participates in the process of EGA. Collectively, our results sum up to a non-canonical role of ZP3 in the oocyte-to-embryo transition in mice. The following discussion is centered on the unexpected cytological location and on the novel functional role assigned to ZP3.

As to the unexpected intracellular embryonic location of ZP3 throughout preimplantation development, we note it resembles the distribution of the proteins of the SCMC, which are located in the subcortex of oocytes and zygotes but are excluded from regions of cell–cell contact in the cleavage-stage mouse embryo (Li *et al.*, 2008). Similar to the reported behavior of the SCMC, ZP3 also redistributed into the area of former cell–cell contact, after mechanical blastomere separation aided by Ca(2+)-free medium. Hints of intracellular ZP localization had been there already in the past, but they were dismissed as error or ignored as trivial cases of carryover from oocyte to embryo. Yet *ZP* mRNAs are present throughout preimplantation, in line with suggestions of neozona formation by preimplantation embryos (reviewed in Denker, 2000). Whether neoformation took place or not was addressed here by performing a live-cell experiment of isotopic protein labeling that has no precedent in preimplantation embryos, although similar work was carried out on postimplantation stages (Calve *et al.*, 2016; Saleh *et al.*, 2019) and in invertebrates (Stoeckius *et al.*, 2014; Beati *et al.*, 2020). We found no evidence of *de novo* ZP protein synthesis during preimplantation, indicating that the *ZP3* gene product detected in the blastocyst was an oocytic protein that perdured in the embryo, without neoformation. Contrary to current understanding of the roles of ZP proteins, ZP3 was more related to the microtubule cytoskeleton than to the secretory vesicles, and was concentrated in the subcortical region of oocytes and zygotes, while it was excluded from regions of cell–cell contact in cleavage-stage mouse embryos. These observations not only challenged the safety of calling some genes ‘oocyte-specific’, but also shaped the choice of the method suited to remove the gene product and see what would happen without it. Clearly, we could not use DNA locus disruption or mRNA interference, since the protein of interest is inherited from the mother without embryonic replenishment; whereas it seemed that we could use an antibody injection approach, since ZP3 was not shielded on the luminal side of the vesicles of the secretory pathway.

As to the functional relevance, ZP3 protein is not only present but it is also functional. This new facet is in line with the report of functional ZP3 found in the germinal vesicles of mouse oocytes (Gao *et al.*, 2017), although the requirement of ZP3 for germinal vesicle breakdown (Gao *et al.*, 2017) is not consistent with previous reports that ZP3−/− oocytes were ovulated and reached the stage of MII (Rankin *et al.*, 2001). Similarly, the functional requirement of ZP3 in our study was earlier (one-cell stage) than previously thought (blastocyst formation by ZP3−/− oocytes; Rankin *et al.*, 2001). The difference between these studies is that our method of gene product inactivation is protein-based and acute, whereas the genetic ablation of the *ZP3* locus during oogenesis gave the oocytes time to elicit compensatory mechanisms (El-Brolosy and Stainier, 2017) until ovulation and fertilization. Testing the function of ZP3 during embryonic development proved more difficult than testing its function during oogenesis. It was made more difficult by the confounder of polyspermic fertilization in the mutational studies of ZP3 conducted at a time when ICSI had yet to become a common tool. In plain words, the ZP3 mutant embryos were crippled with polyploidy. A solution as simple as it was effective was applied by the Dean group, which overcame the problem of polyspermy in ZP3−/− oocytes by performing IVF at low sperm concentration and then selecting the monospermic-fertilized zygotes (Rankin *et al.*, 2001). Until that study came, polyspermy had been regarded as the probable reason why ZP3−/− oocytes failed when fertilized naturally or by conventional IVF, obscuring possible embryonic roles of ZP3 such as the one uncovered here: namely, a requirement for ZP3 during EGA. However, the monospermic-fertilized ZP3−/− oocytes were obtained with a low concentration of wild-type sperm, thereby bringing back through the window (ZP3 allele via sperm) what had been let out of the door (ZP3 locus ablated in oocyte). Based on our results with the microinjection of GFP-tagged ZP3 mRNA into zygotes, we do not feel able to exclude that the paternal ZP3 allele was relevant to the blastocyst formation of the monospermic-fertilized ZP3−/− oocytes. In addition to these considerations, it is also unsafe to compare a phenotype produced by an upstream approach—genetic locus ablation (Rankin *et al.*, 2001)—with a phenotype produced by a downstream approach—protein depletion (this study). Thus, we believe the intracellular embryonic function of ZP3 we described was genuine. This is not the first time a masked phenotype involving ZP3 is uncovered. Upon disruption of the connexin gene *Gja1* (*Connexin43, Cx43*), for example, homozygotic mutant mice died shortly after birth (Roscoe *et al.*, 2001), which precluded their examination at the adult stage. However, when *Cx43−/−* fetal ovary was grafted to wild-type females and allowed to grow, it revealed that mutant oocytes have poorly developed zonae (Ackert *et al.*, 2001), probably because the transzonal projections that normally contain CX43 (Simon *et al.*, 2006) are needed as scaffolding to assemble the zona. Indeed, GJA1 has been detected in the composition of isolated zonae using mass spectrometry (Zhang *et al.*, 2021).

Given the stable and long-lived maternal deposit of ZP3 revealed by the Triton X-100 extraction experiment and by the isotopic labeling experiments, it was clear that the intracellular requirement of ZP3 for embryo survival had to be determined directly in the embryo at the protein level. This was accomplished via methods that either mask the ZP protein or trigger its proteasomal degradation. Live-cell protein masking by antibodies was introduced in the 1980s to show the effects of disrupting the cytokeratin filament network in mouse embryos (Emerson, 1988), while the antibody-based proteasomal degradation of proteins is more recent. Known as ‘*Trim-away*’, it was introduced in 2017 and it has been applied by several laboratories (Clift *et al.*, 2017; Mehlmann *et al.*, 2019; Zhou *et al.*, 2019; Eisa *et al.*, 2020; Gerri *et al.*, 2020; Gassler *et al.*, 2022), including our own (Israel *et al.*, 2019a, 2021), to tackle various proteins of the mammalian oocyte or embryo. As far as we know, target proteins were never chosen among those present within vesicles, probably because it was assumed that *Trim-away* could not possibly work owing to the physical barrier of the membrane. We have shown in this study that ZP3 has a fraction outside the vesicles, and so the problem of the physical barrier does not exist for this target protein. In many *Trim-away* studies, the degradation of target protein was partial (Eisa *et al.*, 2020; Gerri *et al.*, 2020; Jeon and Oh, 2021; Weir *et al.*, 2021), and so it was for our ZP3 as well, yet a knockdown was sufficient to produce a bold effect. In fact, the phenotype of the ZP3 knockdown—pronuclear arrest—was even more severe than that incurred after epitope masking of ZP3: the zygotes not only arrested but also died at the pronuclear stage. Pronuclear arrest is notable, because the mutant phenotypes of genes that are essential for early embryonic development (e.g. maternal-effect genes) typically manifest from the two-cell stage onward (Mitchell, 2022). Next to the case of *Zar1* and its knockout (Wu *et al.*, 2003), the knockdown of ZP3 is one of the few known cases of pronuclear arrest. With this phenotype, it is unlikely that we are dealing with non-specific effects of a particular antibody–antigen choice, since ZP2 and ZP1 subjected to *Trim-away* also resulted in embryopathy. There might be a possibility that the ZP3 antibody used in *Trim-away* cross-reacted with an as yet unidentified protein other than ZP3, since there are various mammalian proteins with a zona pellucida domain (Jovine *et al.*, 2005). Relevant proteins that may come into question—being expressed in the ovary—are the oocyte secreted protein 1 (Oosp1) and the ZP3 paralog Oosp3. However, these proteins are dispensable for female fertility in mice (Abbasi *et al.*, 2020) and have smaller molecular weight than ZP3 (14 and 21 kDa, respectively), which is incompatible with the 75-kDa band detected in our immunoblot (Fig. 2c and Supplementary Fig. S2). Thus, we ascribe our observations to a specific effect on ZP3.

As if the discovery of ZP3’s fourth function were not surprising enough, it has further implications, first and foremost for the compartmentalization of protein synthesis. It is generally accepted that cytosolic proteins are synthesized on free ribosomes, whereas secretory proteins—like ZPs—are synthesized on endoplasmic reticulum-bound ribosomes (Lerner *et al.*, 2003) and end up facing the luminal side of secretory vesicles. The fact that our microinjection of antibody in the ooplasm had an effect means that there must be also a subfraction of ZP3 on the opposite (non-luminal) side of secretory vesicles, i.e. outside of the vesicles and possibly bound in fibrils, consistent with the results of the extraction experiment with Triton X-100 and those of the cytoskeleton disruption experiment with nocodazole. We posit that the ZP3 previously found inside the germinal vesicle of mouse oocytes (Gao *et al.*, 2017) may be to the result of mobilization from fibrils and subsequent translocation into the nucleus. This would also be the origin of the functionally relevant fraction of ZP3 in the context of the experiments conducted here.

To shed light on the molecular bases of the newly discovered function, i.e. intracellular requirement of ZP3 for mouse embryo survival, we subjected the ZP3-knockdown embryos to transcriptome analysis by RNAseq. In order to be sure to measure an effect if present, the analysis was performed 24 h after treatment, although this inevitably meant that the ZP3-knockdown zygotes were arrested at the pronuclear stage while the control zygotes (microinjected with *mCherry-Trim21* mRNA and antibody buffer) had divided. The *Trim-away* embryos were clearly resolved from the controls in PCA, and this was the case also at the midpoint of the one-cell stage, although the gene expression differences were not as pronounced because of the shorter time (10 h instead of 24 h). Gene and MP ontology analysis of the perturbed mRNAs returned ‘cytoplasmic translation’ and ‘nucleolus’ as being among the most enriched terms, which is consistent with a known requirement of protein synthesis (Wang and Latham, 1997) and nucleolar activity (Xie *et al.*, 2022) for the unfolding of the EGA. Indeed, the ZP3-knockdown zygotes incorporated less amino acids in *de novo* protein synthesis, and the detrimental effect of the ZP3 knockdown was confined to the application of *Trim-away* at the time when EGA was in progress. The connection between ZP3 and protein synthesis may raise an eyebrow, yet it is consistent with the assignment of ZP3 to the same cluster as ribosomal proteins in the protein atlas (Uhlen *et al.*, 2005) and in the Harmonizome (Rouillard *et al.*, 2016). The time confinement, whereby EGA had to be in progress in order for the ZP3 knockdown to exert its effect, has been observed also in the case of the orphan nuclear receptor Nr5a2 (Gassler *et al.*, 2022). However, while it was logical to find that a nuclear receptor operated in the EGA, the same cannot be said for a protein hitherto considered to be extracellular. Future research will have to look for the interaction partners of ZP3 in the mouse embryo using co-immunoprecipitation, following the example of germinal vesicle-stage oocytes in which ZP3 was shown to interact with nuclear proteins like PTPRK, AIPL1, and DIAPH2 (Gao *et al.*, 2017). Until then, this is a limitation of our study.

To conclude, the finding that ZP proteins are necessary inside the cell for mouse embryo survival changes the game when it comes to naming gene products that are critical to embryos. Such a finding recruits ZP3 to the family of maternal factors (Innocenti *et al.*, 2022) that are essential for early embryogenesis, at least in mice. It also has implications for what defines a gene as oocyte-specific and for medically assisted reproduction. A high level of transcriptional expression in oocytes is often synonymized with gene specificity or even exclusivity for oocytes (i.e. the gene is expressed nowhere else). However, the protein may outlive the transcriptional silencing, and be abundant up to the blastocyst stage or even beyond. This feature brings together ZP3 and the proteins of the SCMC—a similarity also reinforced by their relationship with the cytoskeleton. How long the proteins outlast the transcripts is an open question, given reports of ZP detection in adult tissues such as testis or in cancer cells (Kraus *et al.*, 2021; Pulawska *et al.*, 2022). Pending cautious extrapolation to other species, our results expand the spectrum of considerations when advising subfertile people on the treatment of subfertility via medically assisted reproduction. When the zona is abnormal, bypassing the problem of polyspermic fertilization (thin zona pellucida) or fertilization failure (thick or hard zona pellucida) may well help the couple to produce embryos; but still these might be unable to produce normal blastocysts owing to the intraembryonic requirement of ZPs uncovered here, reminiscent of the SCMC. Depending on which ZP protein is removed or mutated, this can lead to earlier or later blockade of embryonic cleavage, no matter if the zona defect was bypassed using ICSI.

## Supplementary Material

gaad038_supplementary_data

## Data Availability

All data needed to evaluate this article are provided in the main body or in the Supplementary Materials. Large-scale data (omics) have been deposited in repositories. The RNA-seq dataset of ZP3 *Trim-away* embryos at 10 h has been deposited in the Gene Expression Omnibus of NCBI with accession number GSE232142. The RNA-seq dataset of ZP3 *Trim-away* embryos at 24 h has been deposited in the Gene Expression Omnibus of NCBI with accession number GSE203626. The mass spectrometry dataset of blastocysts obtained after stable isotope labeling with Lys-8 (^13^C_6_$H_{14}^{15}$N_2_O_2_) and Arg-10 (^13^C_6_$H_{14}^{15}$N_4_O_2_) *in vitro* has been deposited in the PRIDE repository (Perez-Riverol *et al.*, 2022) with accession number PXD035570. For convenience, the large-scale data are summarized in Supplementary Tables S1 and S2.
