# Supplementary material for: Intracellular fraction of zona pellucida protein 3 is required for the oocyte-to-embryo transition in mice
Source: Mol Hum Reprod. 2023 Oct 31;29(11):gaad038. doi: 10.1093/molehr/gaad038 (PMC10640839; doi:10.1093/molehr/gaad038)
Supplement: gaad038_supplementary_data [file gaad038_supplementary_data.pdf]

Supplementary information files

Intracellular fraction of zona pellucida protein 3 is required for the oocyte-to-embryo transition in mice

Steffen Israel, Julia Seyfarth, Thomas Nolte, Hannes C.A. Drexler, Georg Fuellen and Michele Boiani

Supplementary Figure S1. Zona-free sample preparation.

Supplementary Figure S2. Uncropped immunoblots of ZP3.

Supplementary Figure S3. Immunofluorescence stainings of zona-intact and zona-free embryos of various preimplantation stages, revealing that the intracellular ZP signal is not limited to ZP3 but applies also to ZP1 and ZP2.

Supplementary Figure S4. Negative control of the ZP3 immunostaining.

Supplementary Figure S5. ZP3 variants.

Supplementary Figure S6. Demonstration of mCHERRY-TRIM21-mediated proteasomal degradation (Trim-away) of the ZP proteins, visualized in live (a, b) or fixed (c) embryos using the mCherry tag of TRIM21.

Supplementary Figure S7. Effect of cytoskeletal disruption by nocodazole and latrunculin B on ZP3 Trim-away.

Supplementary Figure S8. Embryopathy observed after Trim-away of the ZP1 and ZP2 proteins.

Supplementary Figure S9. Allocation of the differentially expressed mRNAs of ZP3-knockdown to predefined EGA gene clusters.

Supplementary Table S1. iBAQ values of blastocysts after 96 h culture for stable isotope labeling of protein synthesis using the non-radioactive isotopic aminoacids Lys-8 ($^{13}C_6H_{14}{}^{15}N_2O_2 \cdot HCl$) and Arg-10 ($^{13}C_6H_{14}{}^{15}N_4O_2 \cdot HCl$). This table is deposited in FigShare.

Supplementary Table S2. RNA sequencing of embryos sampled 24 h after the co-injection of mCherry-Trim21 mRNA with anti-ZP3. Replicates: 3X mCherry-Trim21 mRNA with antibody buffer, 4X ZP3 Trim-away, 1X non-manipulated; dataset GSE203626. This table is deposited in FigShare.

Supplementary Table S3. Mean immunofluorescence intensities measured in the equatorial region of mouse blastomeres (2-cell stage) that incorporated O-propargyl-puromycin after Trim-away of ZP3 vs. control group (OGDB). This table is deposited in FigShare.

Supplementary Table S4. Mean ZP3 immunofluorescence intensities measured in the equatorial region of mouse blastomeres (2-cell stage) that were subjected to Trim-away of ZP3 vs. control group (OGDB). This table is deposited in FigShare.

Supplementary figures

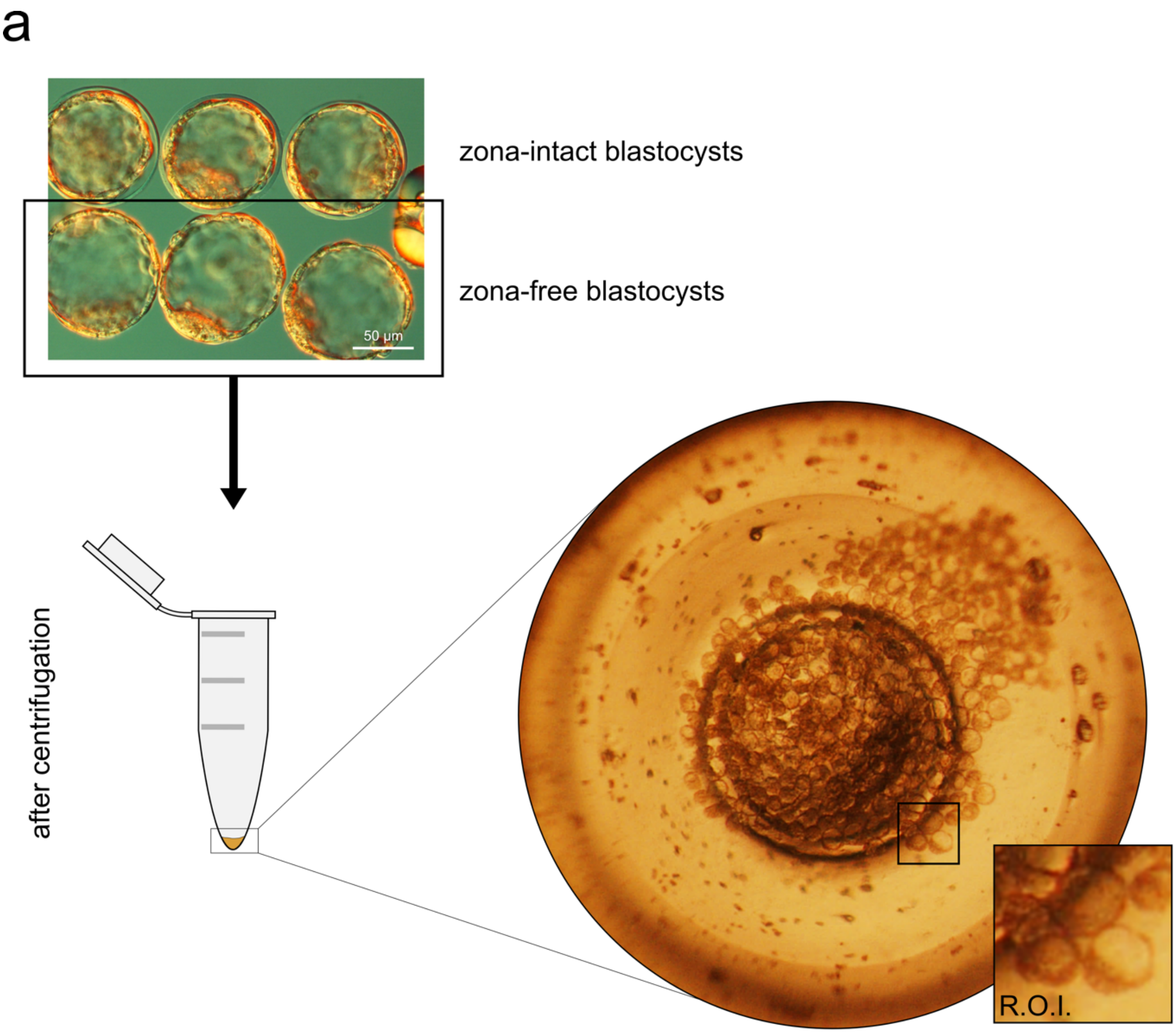

Supplementary Figure S1. Zona-free sample preparation. (a). Zona-free blastocysts prior to lysis for mass spectrometry or immunoblotting. **(b).** Stereomicroscopic view of a pellet of zona-free blastocysts (n ≈ 200) collected on the bottom of a tube after centrifugation, prior to further processing for mass spectrometry or immunoblotting. R.O.I. shows the blastocysts at higher magnification inside the tube. Zona pellucida removal was performed using acidic Tyrode solution. The same procedure described for blastocysts was applied also to oocytes and pre-blastocyst stages. Abbreviations: R.O.I., region of interest.

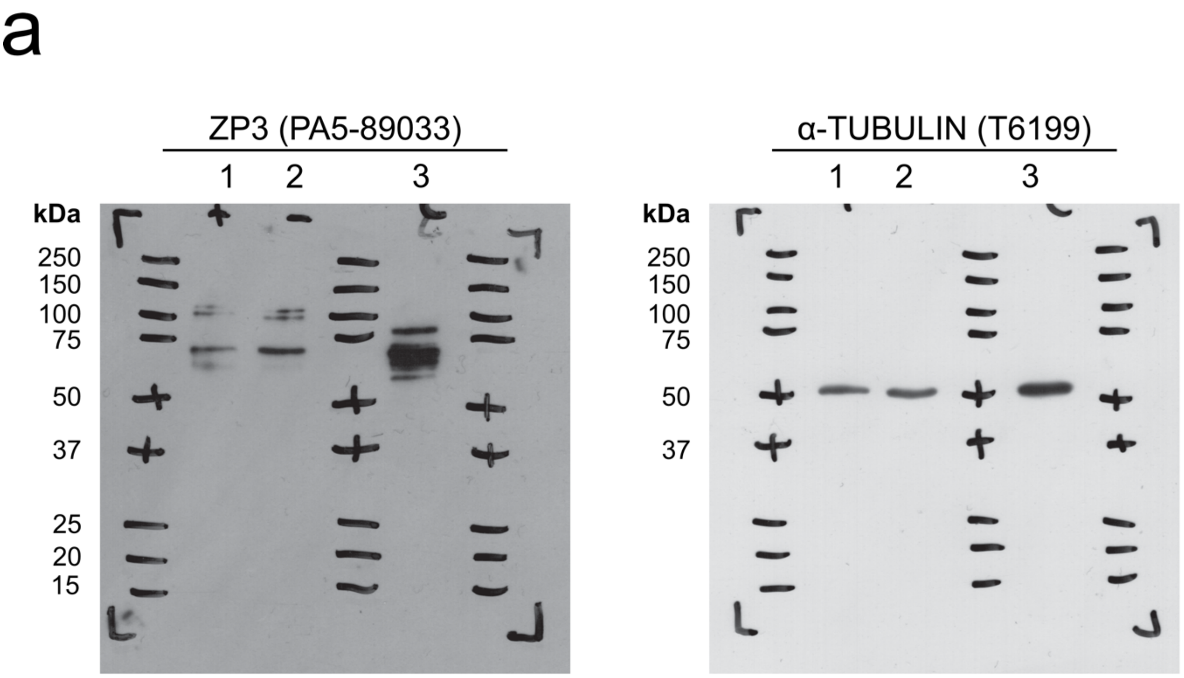

Key
1. #400 non-manipulated blastocysts zona intact
2. #544 non-manipulated blastocysts zona-free
3. 30 µg ES cell protein lysate

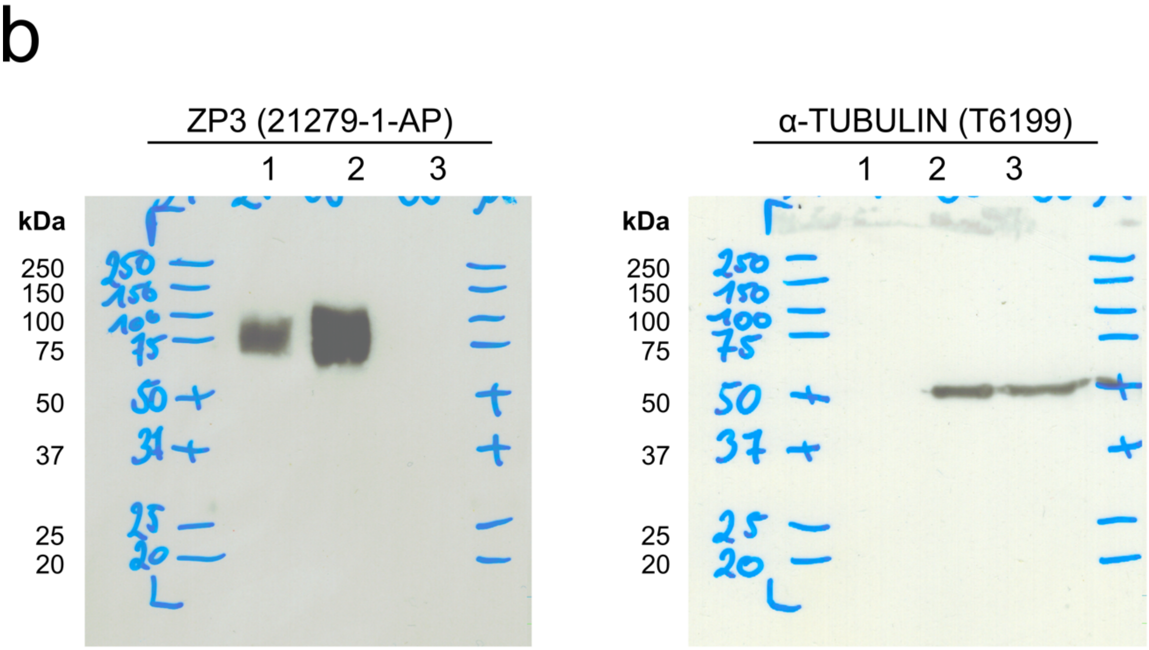

Key
1. #260 Lysed evacuated zonae
2. #260 Intact oocytes
3. 30 µg MEF cell protein lysate

Supplementary Figure S2. Uncropped immunoblots of ZP3. (a). Uncropped immunoblots related to ZP3 in Figure 1C. **(b).** Uncropped immunoblots related to ZP3 in Figure 1E. The same blot in (a) and the same blot in (b) were stripped and reprocessed for α-tubulin as the loading control. The commercial name of antibody is provided in brackets next to ZP3 and α-TUBULIN.

a

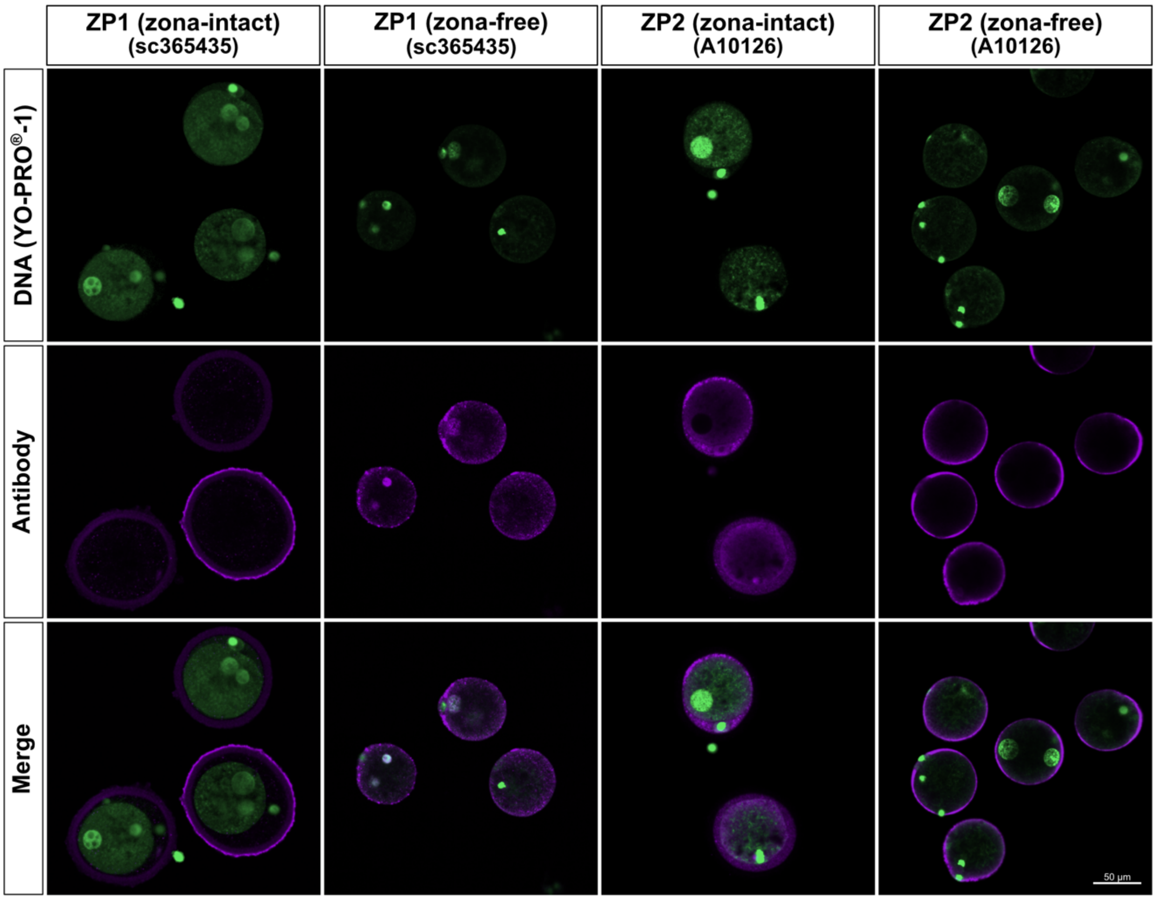

Supplementary Figure S3. Immunofluorescence stainings of zona-intact and zona-free embryos of various preimplantation stages, revealing that the intracellular ZP signal is not limited to ZP3 but applies also to ZP1 and ZP2. (a). Immunofluorescence of ZP1 and ZP2 in zona-free zygotes. Nuclei (DNA) were stained with YO-PRO-1 and are green-fluorescent. The commercial name of antibody is written in brackets underneath the named ZP. Note the extracellular rings present in the samples with intact zonae, whereas removing the zonae causes the rings to disappear, allowing for the intracellular ZP signal to become visible.

a

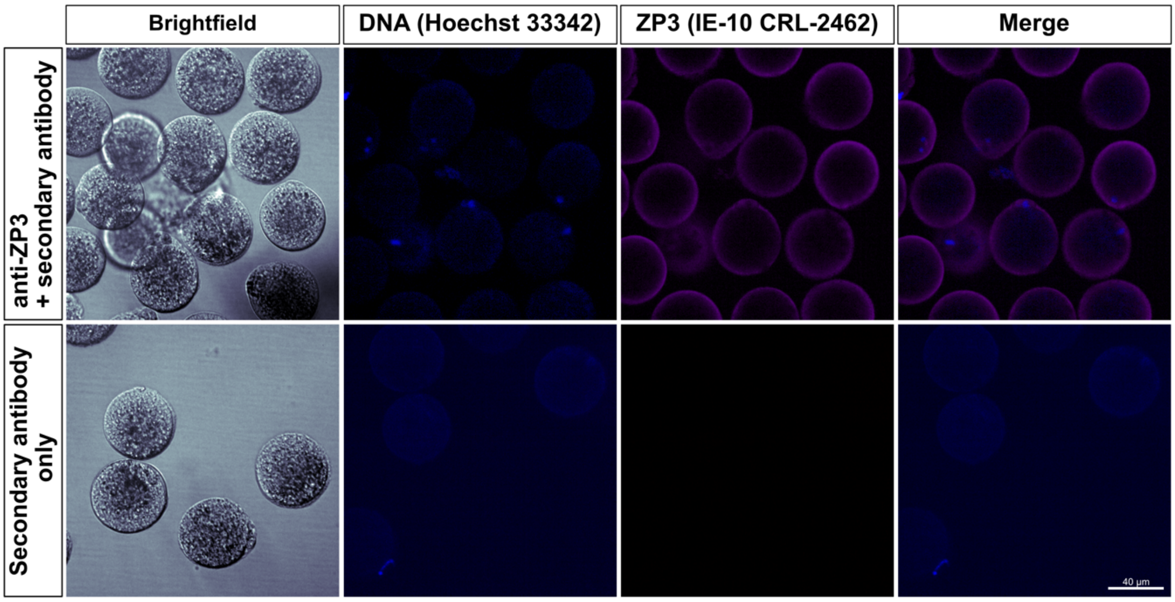

Supplementary Figure S4. Negative control of the ZP3 immunostaining. Metaphase II oocytes were stripped of the zona prior to staining, thereby revealing the intracellular ZP3 (upper row), which could not be seen when the ZP3 antibody was omitted. The commercial name of antibody is written in brackets next to ZP3. DNA was stained with Hoechst 33342 and is blue-fluorescent.

a

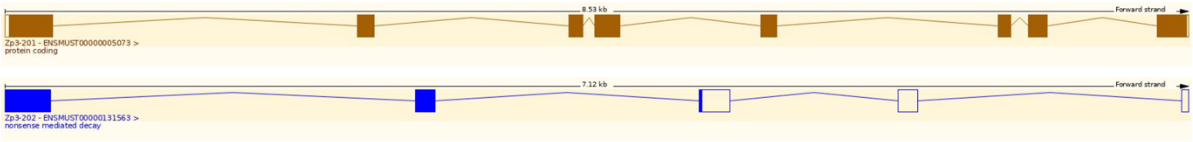

Source: Ensembl

b

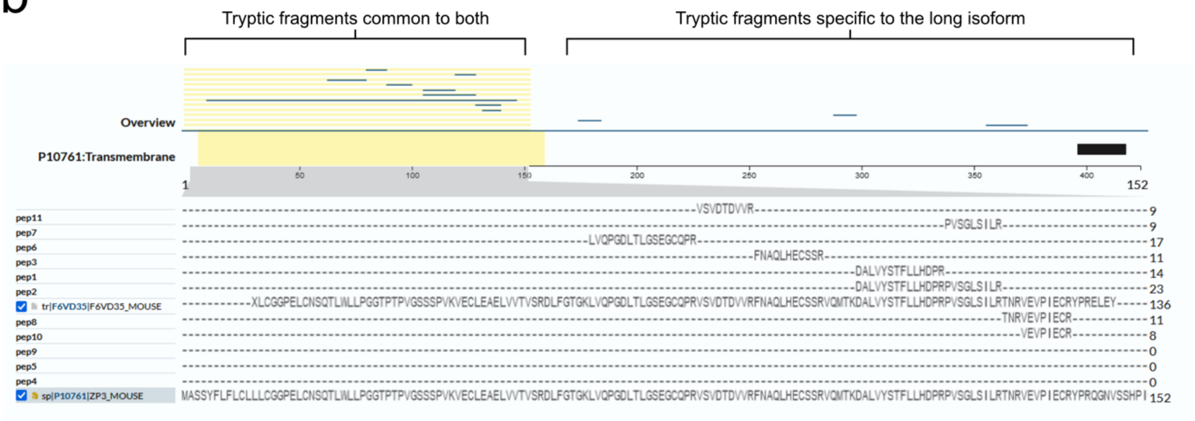

Source: Uniprot

c

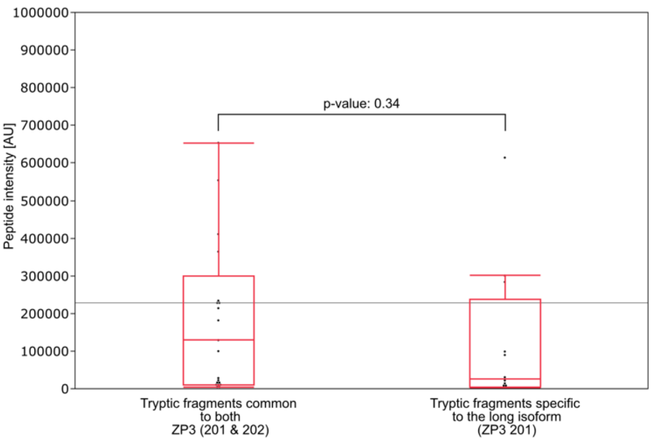

Supplementary Figure S5. ZP3 variants. (a). Alignment of the transcript variants (Ensembl). **(b).** Alignment of the protein variants (Uniprot). **(c).** The intensities of the tryptic fragments of ZP3 produced in mass spectrometry are shown, distinguishing between fragments common to both variants and fragments specific to the long isoform of ZP3 (p-value = 0.34; Wilcoxon test). Abbreviations: AU, Arbitrary Units.

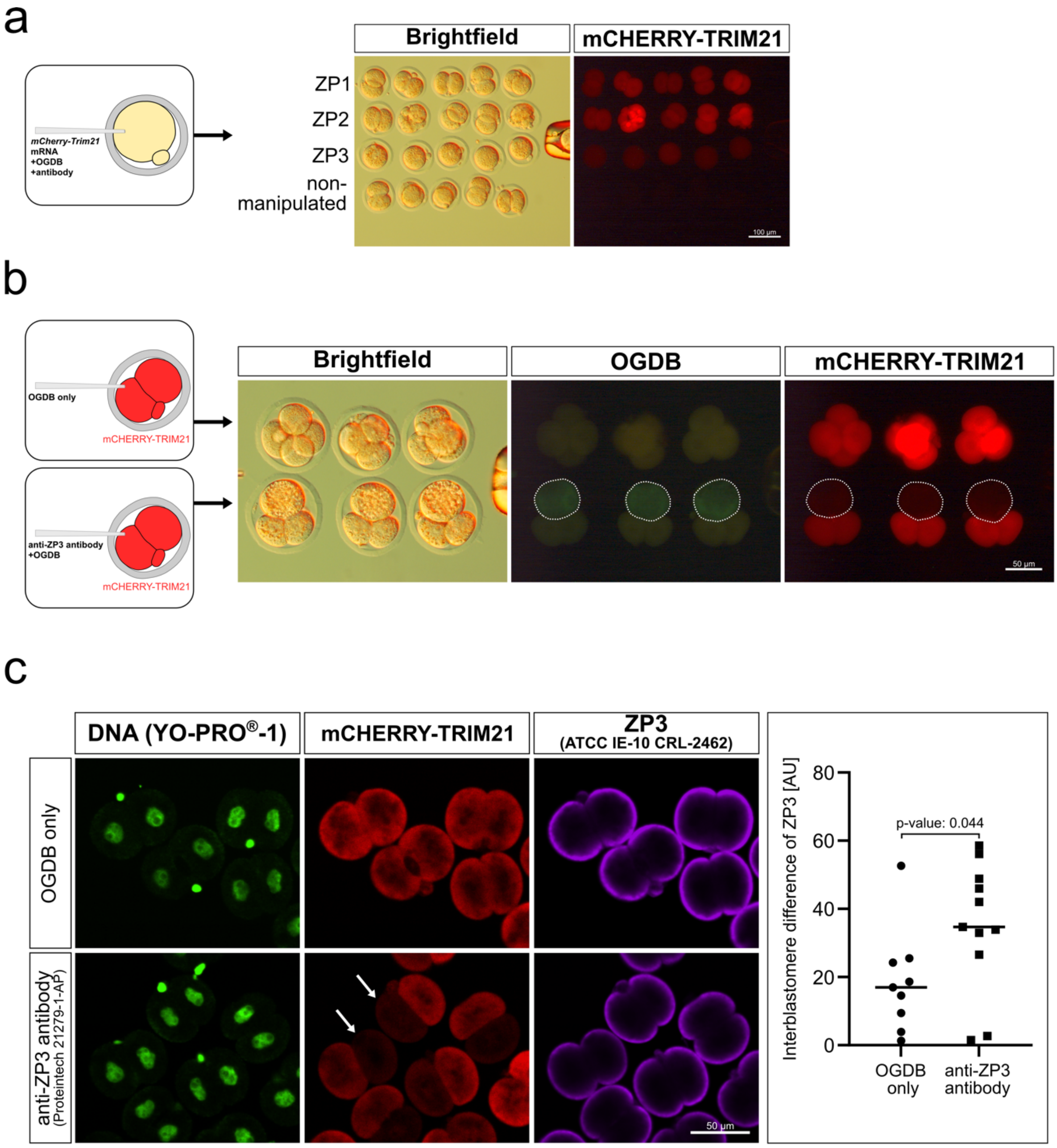

Supplementary Figure S6. Demonstration of mCHERRY-TRIM21-mediated proteasomal degradation (*Trim-away*) of the ZP proteins, visualized in live (a, b) or fixed (c) embryos using the mCherry tag of TRIM21. (a). Bright field and fluorescence images of embryos 24 hours after *Trim-away* of ZP1, ZP2 or ZP3, compared to non-manipulated control. **(b).** Representative images of embryos preloaded with *mCherry-Trim21* mRNA at the 1-cell stage followed by microinjection of anti-ZP3 at the 2-cell stage in one blastomere (note the reduction of mCHERRY fluorescence intensity, dotted circles). **(c).** Demonstration of ZP3 knockdown in 2-cell embryos that were preloaded with *mCherry-Trim21* mRNA at the 1-cell stage and then microinjected at the 2-cell stage with OGDB (control) or with anti-ZP3 (Proteintech 21279-1-AP) in one blastomere only. *Trim-away* achieved a knockdown, as residual ZP3 was still present after the reaction. Residual ZP3 was revealed using the antibody ATCC IE-10 CRL-2462, which binds the ZP3 still left after *Trim-away* operated with Proteintech antibody. Fluorescence intensities were measured in both blastomeres of each embryo, and used to calculate absolute interblastomere differences of ZP3 signal intensity in the *Trim-away vs.* control (OGDB microinjection) groups. Interblastomere differences are higher in the *Trim-away* embryos (p-value = 0.044; Wilcoxon test). The raw measurement data are provided Supplementary Table S4. Nuclei (DNA) were stained with YO-PRO-1 and are green fluorescent. Abbreviations: AU, arbitrary units. OGDB, Oregon green dextran beads, co-injected as tracer. mCherry, fluorescent tag of TRIM21.

a

Effect of latrunculin B and nocodazole during *Trim-away* on the diametral distribution of ZP3
(intensity measured along the oocyte diameter)

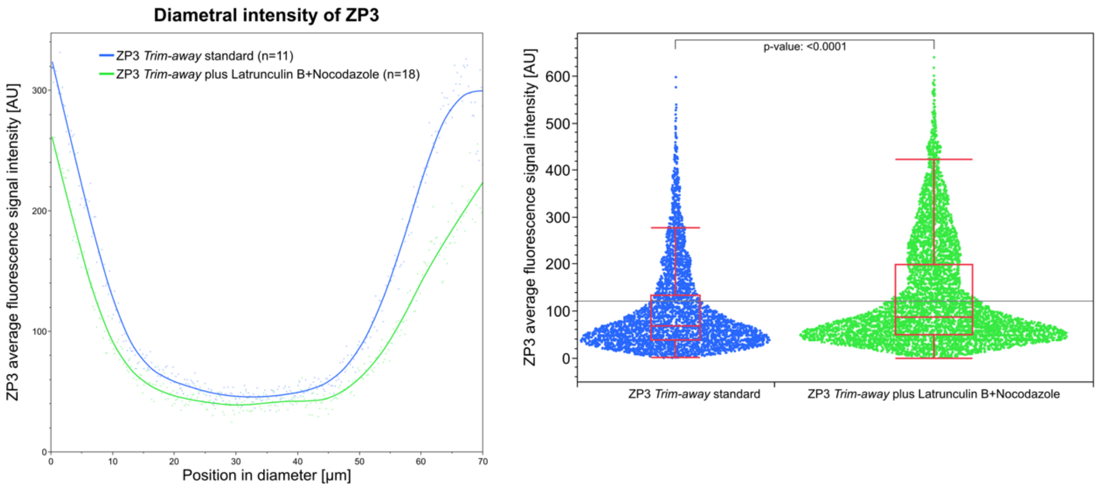

b

Effect of latrunculin B and nocodazole during *Trim-away* on the average intensity of ZP3
(intensity measured in the entire cytoplasm)

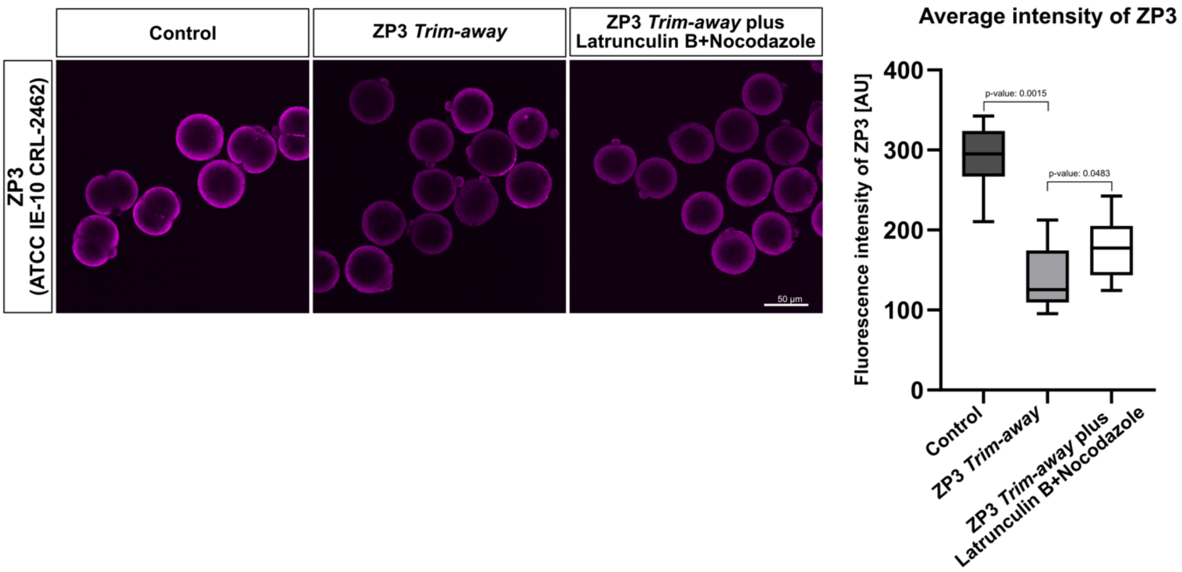

Supplementary Figure S7. Effect of cytoskeletal disruption by nocodazole and latrunculin B on ZP3 *Trim-away*. (a). Average curves of ZP3 immunofluorescence intensity quantified along the oocyte diameter (number, n, of zygotes reported next to treatment). The curves were converted to dot plots for statistical comparison. **(b).** Average values of ZP3 immunofluorescence intensity quantified in the largest cross section of zygotes fixed 10 hours after start of *Trim-away* (number, n, of zygotes reported next to treatment). The values were rendered as box plots for statistical comparison between standard *Trim-away* (n=11) vs. *Trim-away* in combination with cytoskeletal disruption (n=18) vs. dimethylsulfoxide solvent control (n=6). p-values calculated with Wilcoxon test. Abbreviations: AU, arbitrary units.

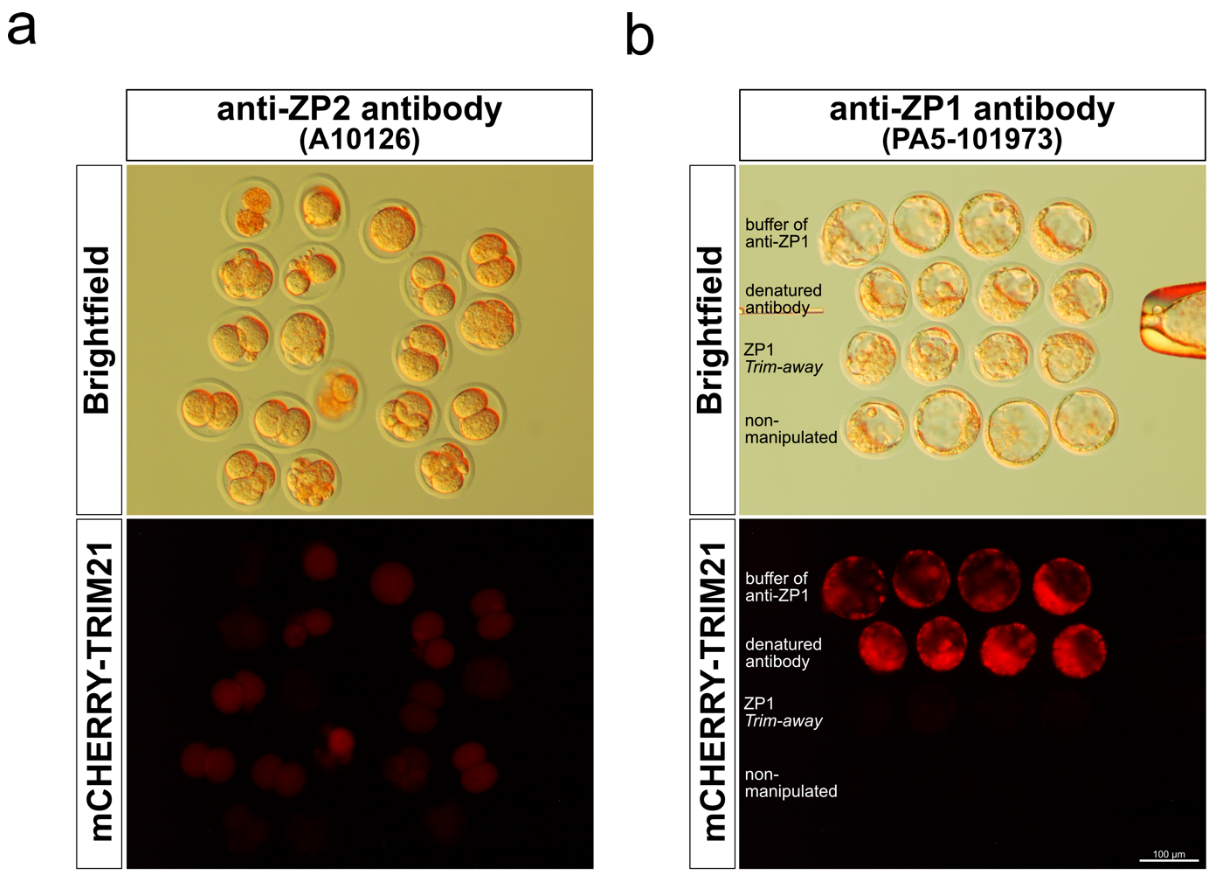

Supplementary Figure S8. Embryopathy observed after *Trim-away* of the ZP1 and ZP2 proteins. (a). Early arrest at the 2-cell or 4-cell stage of zygotes subjected to ZP2 *Trim-away*. **(b).** Stunted blastocyst progression of zygotes subjected to ZP1 *Trim-away*. Note the outwardly normal blastocyst formation in controls (zygotes microinjected with the buffer collected with the 3rd flow of the Amicon filter devices used to purify the antibody; zygotes microinjected with heat-denatured antibody; non-manipulated zygotes). The commercial name of antibody is written in brackets underneath the named ZP. mCherry, fluorescent tag of TRIM21.

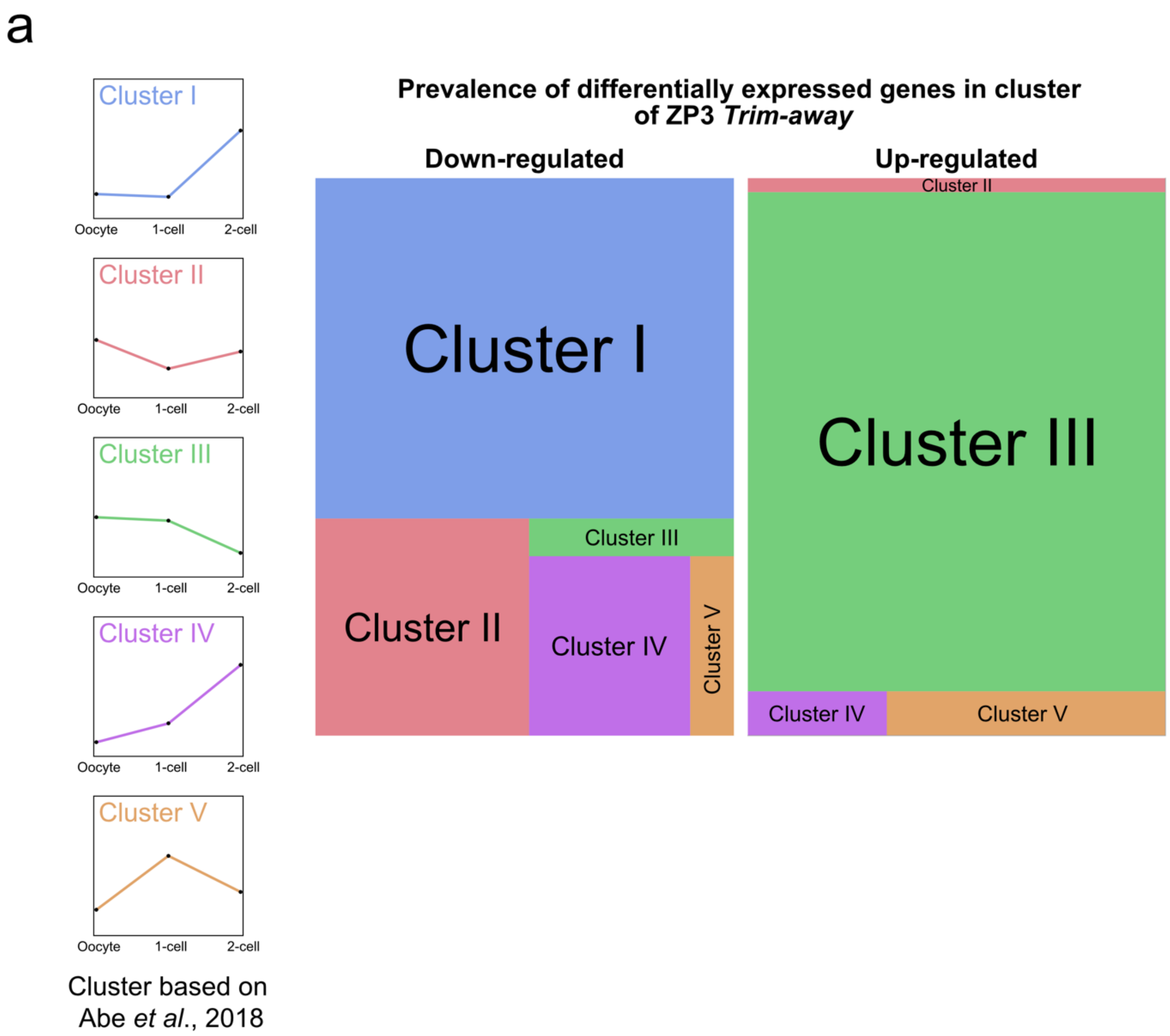

Supplementary Figure S9. Allocation of the differentially expressed mRNAs of ZP3-knockdown to predefined EGA gene clusters. (a). Clusters of EGA-regulated genes as defined by Abe *et al.* (2018) according to the increasing or decreasing mRNA abundance during the progression from oocyte to 2-cell stage. **(b).** Tree maps of the mRNAs differentially expressed after ZP3-knockdown, showing how they relate to the EGA clusters of Abe *et al.* (2018). 'Down-regulated' means that the mRNAs differentially expressed following ZP3-*Trim-away* are less expressed compared to *Trim-only*, while the same mRNAs are up-regulated between the oocyte and 2-cell stage in normal development (cluster I). 'Up-regulated' means that the differentially expressed mRNAs are higher expressed following ZP3-*Trim-away* compared to *Trim-only*, while the same mRNAs are down-regulated between the oocyte and 2-cell stage in normal development (cluster III).

Supplementary tables

Supplementary Table S1:

https://figshare.com/s/880fa87535a1c8193275
DOI: 10.6084/m9.figshare.21205514

Supplementary Table S2:

https://figshare.com/s/f654c959e3bfb7bd304e
DOI: 10.6084/m9.figshare.21205538

Supplementary Table S3:

https://figshare.com/s/5cb24c3773bb2cffbd7c
DOI: 10.6084/m9.figshare.21706982

Supplementary Table S4:

https://figshare.com/s/e84dea5aca70f0a3f564
DOI: 10.6084/m9.figshare.21757148
